# Domain-Aware and Multi-Scale Deep Learning Framework for Sperm Morphology Classification

**DOI:** 10.3390/life16101732

**Published:** 2026-10-15

**Authors:** Imran Gul, Hamza Osman Ilhan, Hakkı Uzun, Merve Huner Yigit, Gorkem Serbes

**Affiliations:** 1 Department of Computer Engineering, Yildiz Technical University, Istanbul 34220,Türkiye; imran.gul@yildiz.edu.tr (I.G.); hoilhan@yildiz.edu.tr (H.O.I.); 2 Department of Urology, Faculty of Medicine, Recep Tayyip Erdoğan University, Rize 53020, Türkiye; hakki.uzun@erdogan.edu.tr; 3 Department of Biochemistry, Faculty of Medicine, Recep Tayyip Erdoğan University, Rize 53020, Türkiye; 4 Department of Biomedical Engineering, Yildiz Technical University, Istanbul 34220, Türkiye; gserbes@yildiz.edu.tr

**Keywords:** sperm morphology classification, ConvNeXt, domain-specific pretraining, supervised contrastive learning

## Abstract

Automated sperm morphology classification can reduce the subjectivity and workload of manual microscopic assessment, but limited benchmark data and subtle morphology-related visual differences remain important challenges. This study proposes a domain-specific ConvNeXt-Tiny-based framework for sperm morphology classification on the SMIDS benchmark. The framework combines Hi-LabSpermMorpho-based domain-specific pretraining, multi-scale feature extraction, attention-guided feature refinement, class-balanced sampling, test-time augmentation, MixUp, label smoothing, focal-loss-based optimization, and supervised contrastive learning. Domain-specific pretraining was compared with ImageNet initialization, and the framework was examined through cumulative ablation, component-removal experiments, and matched-condition architecture comparisons. The best configuration, combining Hi-LabSpermMorpho pretraining with 10× augmented training data, achieved a patch-level average accuracy of 92.73%, numerically the highest among the evaluated configurations and backbones. In matched-condition architecture comparisons, the differences between the proposed framework and EfficientNetV2-S, Swin-T, and domain-pretrained BEiT-Base were not statistically significant. The full training configuration significantly outperformed the baseline configuration under 5× augmentation, whereas the component-removal experiments did not establish the necessity of each individual component. Because the held-out test folds were also used for checkpoint selection, the reported accuracies are subject to selection bias and cannot be regarded as independent test estimates; accordingly, all comparisons should be regarded as exploratory. These findings suggest that domain-specific pretraining can support sperm morphology classification, although independent validation is required.

## 1. Introduction

Male infertility is a major reproductive health concern, and semen analysis remains one of the primary diagnostic procedures used to evaluate male reproductive potential. Among the parameters assessed during semen examination, sperm morphology provides clinically relevant information because abnormalities in sperm head, midpiece, or tail structure may negatively affect fertilization capacity and reproductive outcomes [1,2]. Conventional sperm morphology assessment is generally performed through microscopic visual inspection by trained specialists. Although this procedure is widely used in clinical practice, it is time-consuming, labor-intensive, and vulnerable to intra- and inter-observer variability [3,4]. These limitations have motivated the development of computer-assisted and fully automated approaches that can provide more objective, reproducible, and scalable sperm morphology evaluation [5,6].

Early automated sperm morphology analysis studies generally relied on image processing pipelines based on segmentation, handcrafted feature extraction, and conventional machine learning classifiers. These methods provided important progress toward automated assessment by quantifying morphological characteristics such as sperm head shape, boundary structure, and texture information [7,8]. Related automated sperm analysis studies have also investigated sperm detection, counting, and morphology-oriented preprocessing, highlighting the importance of reliable visual representation before classification [9,10]. However, handcrafted approaches are often sensitive to image quality, staining differences, background artifacts, and preprocessing choices. With the increasing success of deep learning in medical image analysis, convolutional neural networks (CNNs) and transfer learning-based models have become effective alternatives for learning discriminative visual representations directly from sperm images [11].

Several CNN- and ensemble-based studies have reported promising results for sperm morphology classification using public benchmark datasets. These studies include two-stage fine-tuned deep networks, multi-model CNN fusion, ensembled deep learning, and sperm head morphology classification using deep neural networks [12,13]. Additional representative studies have investigated two-stage fine-tuned network fusion, ensemble-based sperm head classification, and deep neural network-based sperm head morphology analysis [14,15,16]. More recent studies have further explored structured learning strategies such as attention-enhanced CNNs, CNN–vision transformer comparisons, knowledge distillation, and multi-level ensemble learning for sperm morphology analysis [17,18]. In particular, recent work has examined CNN–vision transformer performance [19,20], anatomical feature distillation and multi-teacher knowledge distillation [21,22], and category-aware ensemble learning [23].Review-level studies have also emphasized the growing role of deep learning in sperm fertility assessment and morphology-related analysis tasks [24].

Publicly available datasets have played a central role in the systematic evaluation of sperm morphology classification algorithms. In addition to small-scale sperm head morphology benchmarks such as HuSHeM [25], the Sperm Morphology Image Dataset (SMIDS) provides a widely used benchmark for automated sperm morphology classification. SMIDS contains approximately 3000 RGB images labeled as normal sperm, abnormal sperm, and non-sperm [26]. In the present study, SMIDS was selected as the target benchmark dataset because it provides a realistic RGB sperm morphology classification setting with approximately 3000 labeled images acquired using a smartphone-based imaging system. The dataset includes three categories, namely normal sperm, abnormal sperm, and non-sperm, and contains challenging visual conditions such as noise, mixed tails, and multiple sperm heads. These characteristics make SMIDS suitable for evaluating the robustness of the proposed framework in distinguishing morphology-related visual patterns under practical imaging conditions. Although public sperm morphology datasets remain relatively small compared with large-scale natural image datasets, they are valuable for assessing the robustness of automated classification models under limited-data conditions. More recently, Hi-LabSpermMorpho was introduced as a larger expert-labeled sperm morphology dataset with extensive abnormality classes and multiple staining protocols, providing a valuable domain-specific resource for learning sperm morphology-oriented visual representations [27].

Recent advances in attention-based architectures and vision transformers have also influenced medical image analysis and sperm morphology classification. The transformer architecture was originally introduced for sequence modeling, and vision transformer models later extended this idea to image recognition by representing images as patch sequences [28,29]. Subsequent transformer variants, including BEiT and DeiT, further improved self-supervised visual representation learning and data-efficient transformer training for image recognition tasks [30,31]. In medical imaging, transformer-based models have attracted increasing attention because they can capture long-range spatial dependencies and global shape-related information [32]. A recent comparative benchmark evaluated multiple vision transformer variants for sperm morphology classification and reported strong results with BEiT-Base, achieving 92.50% accuracy on SMIDS [20]. This result provides an important reference point for assessing whether a ConvNeXt-Tiny-based convolutional architecture, when supported by sperm-domain pretraining and robust training strategies, can achieve competitive performance against transformer-based approaches.

Despite the strong performance of transformer-based models, convolutional architectures remain highly relevant for biomedical image classification because their inductive biases support the learning of local texture, contour, and morphology-related patterns. Modern CNN families have revisited conventional convolutional design principles and demonstrated that carefully designed convolutional models can remain competitive with transformer-based architectures while offering practical advantages in computational efficiency and training stability [33]. However, models trained on limited biomedical datasets remain vulnerable to overfitting and poor generalization. Class-balanced sampling, data augmentation, and label smoothing have therefore been widely investigated as strategies for improving robustness under limited-data and class-imbalanced conditions [34,35].

To address these challenges, this study proposes a ConvNeXt-Tiny-based sperm morphology classification framework that combines domain-specific pretraining with multi-scale, attention-guided feature extraction. ConvNeXt is a modern convolutional architecture developed by revisiting and updating standard CNN design principles in light of recent advances in vision models [33]. In the proposed framework, features extracted from the early, middle, and late stages of ConvNeXt-Tiny capture complementary low-level texture, mid-level structural, and high-level semantic morphology information. These features are refined using attention modules and integrated into a unified representation for classification. The training pipeline further incorporates class-balanced sampling, test-time augmentation, MixUp, label smoothing, focal-loss-based optimization, and supervised contrastive learning to improve robustness under limited-data conditions.

The primary objective of this study is to evaluate whether sperm-domain pretraining and the proposed multi-scale learning framework can improve classification performance on the SMIDS benchmark dataset. Accordingly, Hi-LabSpermMorpho-based domain-specific pretraining is compared with ImageNet initialization across different SMIDS augmentation settings. A cumulative ablation study is also conducted by progressively incorporating class-balanced sampling, test-time augmentation, attention-based feature refinement, MixUp, label smoothing, and supervised contrastive learning into the baseline configuration. This design enables the cumulative effect of the evaluated components to be examined under different initialization and augmentation conditions.

Overall, this study provides a structured evaluation of a ConvNeXt-Tiny-based convolutional framework for automated sperm morphology classification. Its main contribution lies in integrating sperm-domain representation learning with multi-scale attention-guided features and robust training strategies, while systematically examining their combined behavior on SMIDS. The resulting framework is evaluated against previously reported SMIDS benchmarks to determine whether a domain-aware ConvNeXt-Tiny model can achieve competitive performance relative to recent transformer-based approaches.

## 2. Materials and Methods

### 2.1. Datasets

This study employed the publicly available Sperm Morphology Image Dataset (SMIDS) as the target benchmark dataset to assess the performance of the proposed automated sperm morphology classification model [14,26]. SMIDS was selected because its imaging characteristics and class structure provide a challenging and application-oriented setting for automated sperm morphology classification. The dataset was acquired using a smartphone-based imaging system and contains approximately 3000 RGB images with three annotated categories: normal sperm, abnormal sperm, and non-sperm. The inclusion of a non-sperm category is methodologically important because fully automated morphology analysis systems must not only distinguish normal and abnormal sperm cells, but also reject visually similar non-target structures that may appear in microscopic fields. In addition, SMIDS contains realistic image-level difficulties, including noise, mixed tails, and multiple sperm heads, which increase intra-class variability and make the classification task more representative of practical, less manually standardized imaging conditions. These properties make SMIDS an appropriate target-domain benchmark for evaluating whether the proposed framework can learn robust morphology-related representations and remain stable under different augmentation, initialization, domain-specific pretraining, and ablation configurations [20].

Because the proposed framework transfers representations learned from a sperm morphology source domain to the SMIDS target domain, visual compatibility between the source and target datasets is an important methodological consideration. In sperm morphology classification, this compatibility is influenced not only by the biological similarity of sperm structures, but also by image acquisition conditions, staining appearance, color and contrast distribution, and the presence of morphology-related visual patterns. Therefore, the domain-specific pretraining stage was designed to adapt ImageNet-pretrained ConvNeXt-Tiny weights toward sperm-domain visual representations before SMIDS fine-tuning [20,27].

In addition, a clinical sperm morphology dataset, referred to as Hi-LabSpermMorpho, was used only as a domain-specific pretraining source. Hi-LabSpermMorpho consists of the BesLab, Histoplus, and GBL subsets and was introduced as an expert-labeled sperm morphology dataset with extensive abnormality classes and clinical staining variability [27]. In this study, Hi-LabSpermMorpho was used to provide sperm morphology-oriented initialization before fine-tuning the proposed ConvNeXt-Tiny framework on the target SMIDS benchmark dataset. A summary of the datasets employed in this study is listed in Table 1.

#### 2.1.1. SMIDS Benchmark Dataset

The Sperm Morphology Image Dataset (SMIDS) was introduced for automated sperm morphology analysis and has been widely used in sperm morphology classification studies. SMIDS was created from stained microscopic ocular images acquired using a smartphone-based imaging approach mounted on the ocular part of a microscope. In the original data acquisition protocol, semen samples were stained using a modified hematoxylin–eosin assay before image acquisition to improve the visualization of sperm morphological structures. The images were acquired using an Olympus BX50 microscope (Olympus Optical Co., Ltd., Tokyo, Japan) with 20× magnification, a 12 V/100 W halogen illumination system, an image resolution of 3890 × 3000 pixels, and an object-space resolution of 1 μm/pixel [26].

The dataset was generated from 200 stained ocular images collected from 17 subjects. Candidate sperm and non-sperm regions were first extracted from the full microscopic images using an automated segmentation and cropping pipeline, and the resulting image patches were then manually validated and labeled by experts. The final SMIDS dataset contains 3000 RGB image patches with an approximate image size of 190 × 170 pixels. The images are categorized into three classes: normal sperm, abnormal sperm, and non-sperm. According to the dataset distribution reported in previous benchmark studies, SMIDS includes 1021 normal sperm images, 1005 abnormal sperm images, and 974 non-sperm images [20,26].

**Figure 1:**
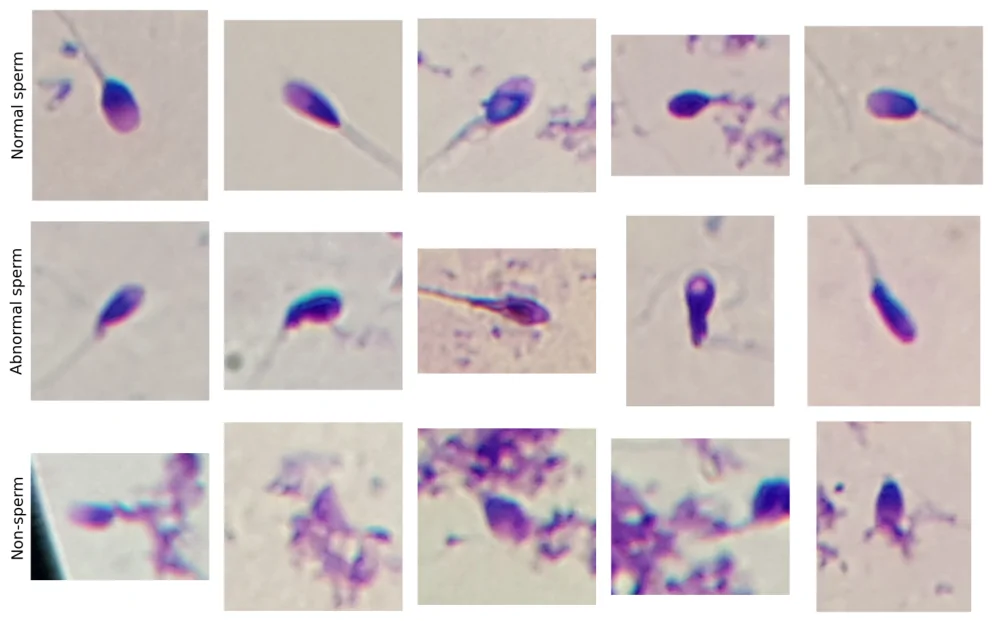
Randomly selected sample images from the SMIDS dataset for the normal sperm, abnormal sperm, and non-sperm classes.

An important characteristic of SMIDS is that it reflects practical microscopic imaging conditions rather than a fully manually standardized sperm-head-only setting. The dataset includes stained sperm morphology images acquired through a low-cost smartphone-based microscope setup and contains realistic visual challenges such as noise, mixed tails, multiple sperm heads, and non-sperm stained blobs. In addition, the abnormal sperm class is represented as an aggregate morphology category rather than being divided into fine-grained abnormality subtypes. These properties make SMIDS a suitable target-domain benchmark for evaluating whether the proposed framework can learn robust morphology-related representations under realistic acquisition variability and distinguish normal sperm, abnormal sperm, and visually similar non-sperm regions [20,26]. Following the evaluation protocol of the original SMIDS study [26], a patch-level five-fold cross-validation was used, in which 80% of the patches were used for training and 20% for testing in each fold, and every patch was tested exactly once. Because the publicly available SMIDS patches do not include subject or source ocular image identifiers, subject-level or source-image-level grouping could not be applied. Consequently, patches originating from the same subject, source image, or microscopic field may be distributed across different folds. To examine one aspect of this dependence, the patches were screened for exact and near-duplicate images across folds using pixel-level hashing and perceptual image hashing. A total of 82 images (2.73%) had a duplicate or near-duplicate in a different fold; after excluding these images, the accuracy of the best configuration remained unchanged (92.73% on the remaining 2918 images), indicating that the reported accuracy was not driven by exact or near-duplicate image overlap. However, this analysis does not exclude dependence between different patches from the same subject or microscopic field; therefore, the reported results reflect patch-level benchmark performance rather than generalization to unseen subjects. All compared models were evaluated on the same folds. Representative sample images of the three SMIDS classes are shown in Figure 1.

#### 2.1.2. Hi-LabSpermMorpho Pretraining Dataset

**Figure 2:**
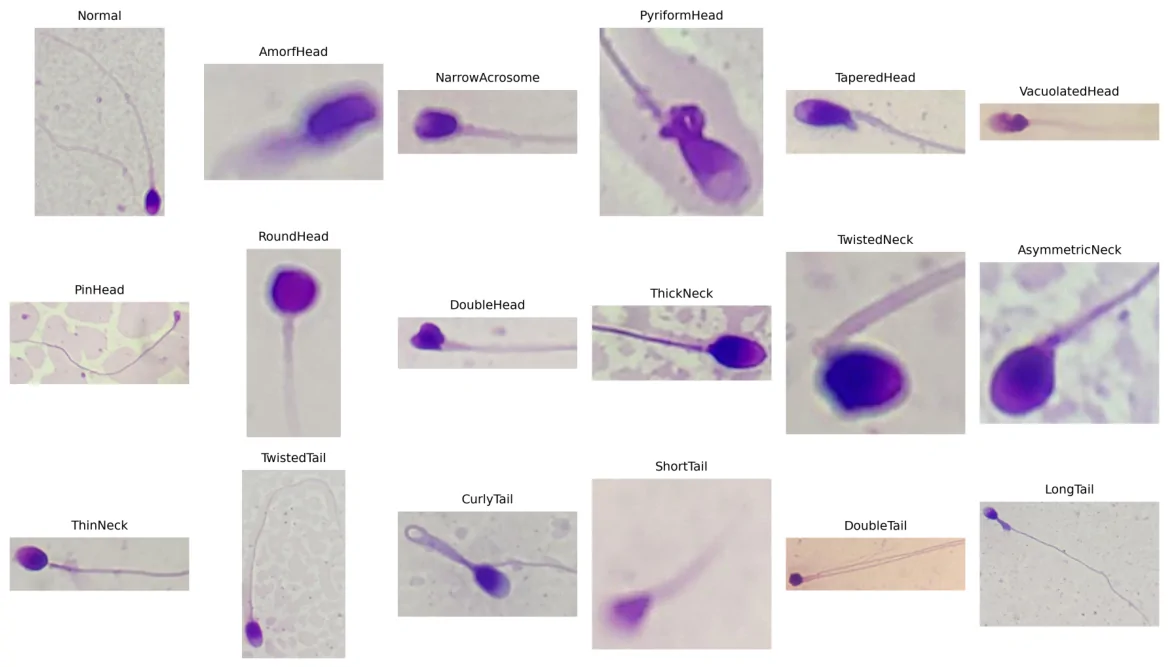
Randomly selected sample images from Hi-LabSpermMorpho, one for each of the 18 morphological classes, ordered as normal, head, neck/midpiece, and tail abnormalities. The images originate from the three staining kits and therefore differ in color and patch size.

To investigate the benefit of domain-specific pretraining, the ImageNet-pretrained ConvNeXt-Tiny model was further pretrained on Hi-LabSpermMorpho before SMIDS fine-tuning. Hi-LabSpermMorpho consists of the BesLab, Histoplus, and GBL subsets and was used as a sperm morphology source-domain dataset to adapt the model toward sperm-related visual representations prior to evaluation on the SMIDS target benchmark. Hi-LabSpermMorpho contains 49.345 sperm morphology images distributed across 18 morphological classes. Hi-LabSpermMorpho is an expert-labeled dataset designed to represent detailed sperm morphology categories and clinical staining variability [27]. In this study, Hi-LabSpermMorpho was used only for domain-specific pretraining and was not included in the final testing stage. The pretrained ConvNeXt-Tiny weights obtained from this dataset were used to initialize the proposed framework before fine-tuning on SMIDS. This design allowed us to evaluate whether sperm morphology-oriented pretraining improves downstream SMIDS classification performance compared with ImageNet initialization, which is widely used as a general-purpose visual pretraining strategy in deep learning-based image recognition [36]. Hi-LabSpermMorpho contains 49,345 expert-labeled RGB sperm morphology patches acquired using three different Diff-Quick staining methods (BesLab, Histoplus, and GBL) [27]. The images were acquired from 1001 patients at 100× magnification under bright-field microscopy (ZEISS AXIO LAB A1, Carl Zeiss Microscopy GmbH, Jena, Germany; Olympus BX43, Olympus Corporation, Tokyo, Japan) using a mobile phone mounted on the microscope ocular, and each sperm cell was labeled by two experts according to WHO criteria. The dataset contains 18 morphological classes comprising one normal class and 17 abnormality classes covering three anatomical regions of the spermatozoon: eight head abnormalities, four neck/midpiece abnormalities, and five tail abnormalities. The class distribution is highly imbalanced, ranging from 7968 images for AmorfHead to 135 images for DoubleHead (Table 2); a sample image of each class is shown in Figure 2.

Domain-specific pretraining was formulated as a supervised 18-class classification task on the combined images of the three staining kits. ConvNeXt-Tiny was initialized with ImageNet-pretrained weights, the final classification layer was replaced with an 18-class output layer, and all parameters were trainable. The training and test partitions of the dataset were merged, and a random 20% of the images were held out as a validation set (random seed 42); no images were reserved for testing, because the purpose of this stage was representation learning rather than performance evaluation. The model was trained using the cross-entropy loss and the AdamW optimizer (learning rate $1\times 10^{-4}$, weight decay $1\times 10^{-4}$) with cosine annealing, a batch size of 16, and at most 50 epochs. Early stopping was based on validation accuracy, and the checkpoint with the highest validation accuracy was retained. No class-rebalancing strategy was applied during pretraining. Hi-LabSpermMorpho and SMIDS are independent datasets: they were collected at different institutions (Recep Tayyip Erdoğan University, Rize, and Istanbul University, respectively), from different patient cohorts (1001 and 17 patients), using different staining protocols (Diff-Quick and modified hematoxylin–eosin) and different microscopes; therefore, no image or patient overlap exists between the source and target datasets. After domain-specific pretraining, the 18-class classification head was discarded and only the learned ConvNeXt-Tiny backbone weights were transferred to the SMIDS model. No direct class-to-class mapping was performed between the two datasets. Instead, Hi-LabSpermMorpho pretraining was used to provide morphology-sensitive feature representations before adaptation to the three-class SMIDS task. During SMIDS fine-tuning, the early ConvNeXt stage was kept frozen to preserve the transferred low-level representations, while the middle and late stages were trainable and adapted to the target task.

### 2.2. Proposed Framework

**Figure 3:**
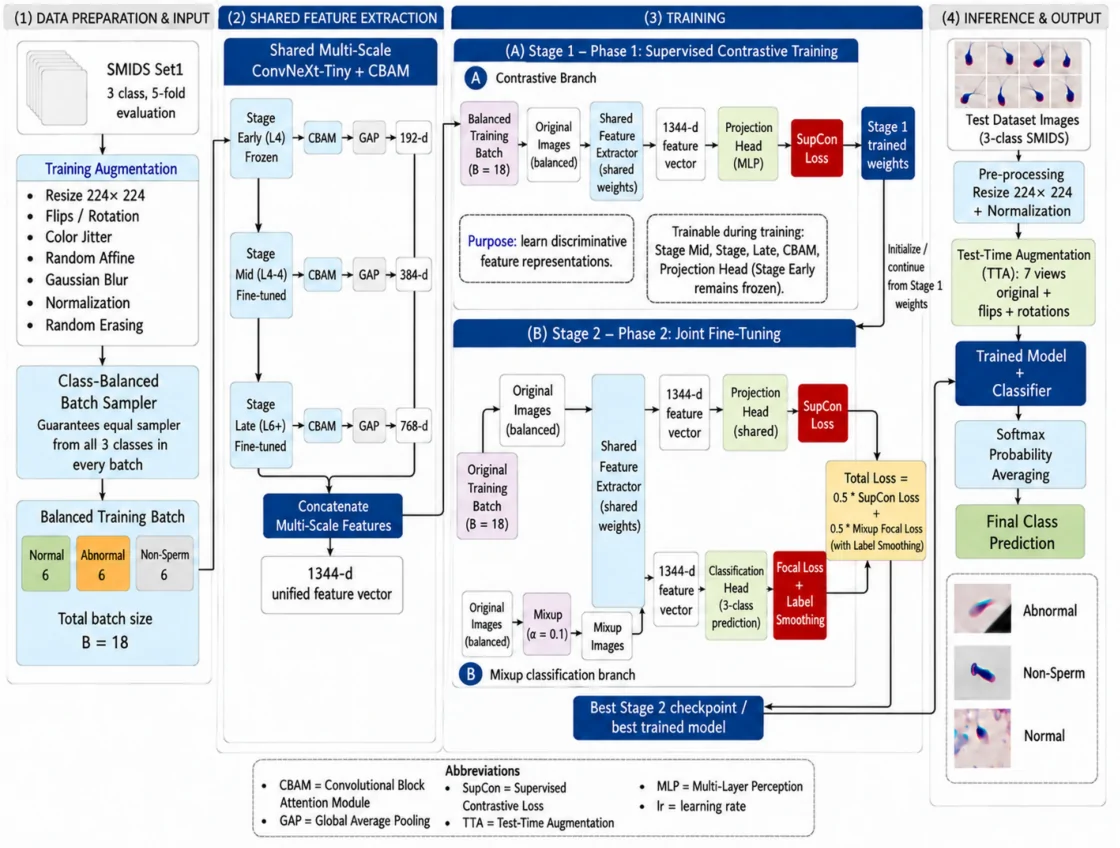
Overview of the proposed ConvNeXt-Tiny-based sperm morphology classification framework. The pipeline consists of data preparation, domain-specific model initialization, multi-scale feature extraction, training and optimization, and inference. Raw microscopic images are resized, normalized, augmented, and sampled using a class-balanced strategy. The proposed framework evaluates two initialization routes: direct fine-tuning from ImageNet-pretrained ConvNeXt-Tiny weights and additional Hi-LabSpermMorpho domain-specific pretraining before SMIDS fine-tuning. In the proposed domain-specific route, ImageNet-pretrained ConvNeXt-Tiny weights are further adapted on Hi-LabSpermMorpho and then transferred to the SMIDS target benchmark. Multi-scale ConvNeXt-Tiny features are extracted from early, middle, and late stages, refined using attention modules, pooled, and concatenated into a 1344-dimensional feature representation. The unified representation is used by both the classification branch and the supervised contrastive learning branch. During inference, test-time augmentation and softmax probability averaging are used to obtain the final SMIDS class prediction.

The proposed framework was designed to improve sperm morphology classification on the SMIDS benchmark dataset by integrating domain-specific pretraining, multi-scale feature extraction, attention-based feature refinement, and complementary training strategies within a ConvNeXt-Tiny-based architecture. As illustrated in Figure 3, the pipeline comprises four main blocks: data preparation and input, model initialization and core multi-scale feature extraction, training and optimization, and inference and output. These blocks and their associated modules are described in detail in the following subsections.

In the data preparation and input block, microscopic sperm images are resized to 224×224 pixels and normalized before being passed to the network. Training augmentation is applied to increase sample diversity and reduce overfitting in the limited-data setting [35]. A class-balanced sampler then constructs mini-batches containing equal numbers of samples from the normal sperm, abnormal sperm, and non-sperm classes. In addition to reducing class dominance during optimization, balanced mini-batches provide sufficient same-class samples for constructing positive pairs during supervised contrastive learning [34,37].

Before SMIDS fine-tuning, the proposed framework considers two model initialization routes. In the ImageNet initialization route, ConvNeXt-Tiny is initialized with ImageNet-pretrained weights and directly fine-tuned on SMIDS. In the domain-specific route, the same ImageNet-pretrained ConvNeXt-Tiny model is first further pretrained on Hi-LabSpermMorpho to adapt the model toward sperm morphology-related visual representations, and the resulting checkpoint is then fine-tuned on SMIDS. Therefore, Hi-LabSpermMorpho pretraining constitutes the domain-specific initialization component of the proposed framework.

The core multi-scale feature extraction block employs the ConvNeXt-Tiny backbone obtained from the selected initialization route. ConvNeXt incorporates modern convolutional design principles while preserving the locality and efficiency advantages of convolution-based feature extraction [33]. Feature maps are extracted from the early, middle, and late backbone stages to capture complementary texture-level, structural, and semantic morphology information. Each feature map is refined using an attention module based on the convolutional block attention mechanism and transformed into a one-dimensional vector through global average pooling [38]. The resulting 192-, 384-, and 768-dimensional vectors are concatenated to form a unified 1344-dimensional representation shared by the classification and supervised contrastive learning paths.

The training and optimization block consists of two sequential stages. During Stage 1, the original class-balanced images and their class labels are used for supervised contrastive training to encourage tighter same-class clustering and to increase the separation between different-class embeddings in the learned feature space [37]. The parameters obtained at the end of Stage 1 are subsequently used to initialize Stage 2 joint fine-tuning; no checkpoint selection is performed in Stage 1. During Stage 2, the shared multi-scale feature extractor, classification head, and contrastive branch are jointly optimized. MixUp is applied online only to the Stage 2 classification input stream, whereas the supervised contrastive objective continues to use the original non-mixed images and class labels [39]. Label smoothing, focal-loss-based optimization, AdamW, learning-rate scheduling, and early stopping are additionally incorporated to improve generalization and optimization stability under limited-data conditions [40,41,42,43].

In the inference and output block, the best-performing Stage 2 checkpoint is used to classify the SMIDS test images. Test-time augmentation evaluates seven versions of each image: the original image, horizontal and vertical flips, rotations of $90^{\circ }$, $180^{\circ }$, and $270^{\circ }$, and a transpose transformation. The softmax probability vectors obtained from these variants are averaged, and the class with the highest mean probability is selected as the final prediction. The complete test-time augmentation procedure is described in Section Test-Time Augmentation and Final Prediction.

#### 2.2.1. Data Preparation and Input

The data preparation and input block transforms the SMIDS microscopic images into standardized and class-balanced mini-batches before they are passed to the shared multi-scale feature extraction network. As illustrated in Figure 3, this block comprises input preprocessing, training-time augmentation, and class-balanced batch construction.

##### Input Preprocessing and Training Augmentation

All SMIDS images were resized to 224×224 pixels, converted into tensors, and normalized using the standard ImageNet channel-wise mean values of [0.485,0.456,0.406] and standard deviation values of [0.229,0.224,0.225]. This normalization scheme was used to maintain compatibility with the ImageNet-pretrained ConvNeXt-Tiny initialization used in both the direct ImageNet initialization route and the Hi-LabSpermMorpho domain-specific pretraining route. During training, online data augmentation was applied to increase sample diversity and reduce overfitting, which is particularly important in limited-data biomedical image classification settings [35]. The augmentation pipeline included random horizontal and vertical flipping, random rotation within $\pm 20^{\circ }$, brightness, contrast, saturation, and hue variations, random affine translation and scaling, Gaussian blurring, and random erasing. Specifically, the affine transformation used translations of up to 10% of the image dimensions and a scaling range of 0.9–1.1, whereas random erasing was applied with a probability of 0.15. These stochastic transformations were applied only to the training images. Test images were resized, converted into tensors, and normalized without training-time augmentation. Following preprocessing and augmentation, the training samples were passed to the class-balanced batch sampler.

##### Class-Balanced Sampling

Class-balanced sampling was applied during mini-batch construction to ensure equal representation of the three SMIDS classes. In addition to reducing class dominance during optimization, this strategy guarantees that each mini-batch contains sufficient same-class samples for constructing positive pairs in supervised contrastive learning [34]. Let *K* denote the number of classes and *n* denote the number of samples selected from each class. The balanced batch size *B* was defined as

(1) B=K×n.

For the three-class SMIDS dataset, comprising normal sperm, abnormal sperm, and non-sperm images, six samples were selected from each class (n=6). Therefore, each class-balanced mini-batch contained

(2) B=3×6=18

samples. The value of six samples per class was selected as a practical mini-batch design to provide sufficient same-class examples for supervised contrastive learning while maintaining compatibility with GPU memory constraints. This setting was fixed before the final evaluation and was not selected based on test-set performance, thereby avoiding test-set-driven hyperparameter selection.

The sample indices within each class were randomly shuffled, and the selected samples were subsequently shuffled within each mini-batch before being passed to the shared feature extraction network. This procedure maintained equal class representation while ensuring that multiple samples belonging to the same class were available in every mini-batch for supervised contrastive optimization.

#### 2.2.2. Core Multi-Scale Feature Extraction

The core multi-scale feature extraction block generates a unified representation by processing SMIDS images through a partially fine-tuned ConvNeXt-Tiny backbone and extracting complementary features from its early, middle, and late stages. As illustrated in Figure 3, the selected feature maps are refined using attention modules, transformed into one-dimensional vectors through global average pooling, and concatenated before being passed to the training branches.

##### ConvNeXt-Tiny Backbone

ConvNeXt-Tiny was selected as the backbone architecture because it provides a modern convolutional design with strong representation learning capacity and preserves the locality advantages of convolution-based feature extraction, making it a suitable convolutional alternative to transformer-based architectures for SMIDS classification [33]. ConvNeXt modernizes standard convolutional neural network design by incorporating architectural choices inspired by recent vision models, while preserving the efficiency and locality advantages of convolution-based feature extraction. This design choice is consistent with the broader trend of developing efficient convolutional architectures that remain competitive in image recognition, as demonstrated by EfficientNetV2 and MobileNet-based models [26,44]. Modern deep convolutional architectures also commonly rely on regularization mechanisms, such as stochastic depth, to improve optimization stability and generalization in deep networks [45]. Therefore, ConvNeXt-Tiny provides a suitable backbone for limited-data biomedical image classification tasks, where both discriminative representation learning and computational efficiency are important.

In this study, the ConvNeXt-Tiny backbone was initialized using two different strategies depending on the experimental setting. In the general-domain setting, the model was initialized using ImageNet-pretrained weights and then fine-tuned on SMIDS. ImageNet pretraining is widely used as a general-purpose visual initialization strategy for deep neural networks because it provides transferable low-level and mid-level visual representations learned from a large-scale image dataset [36]. In the domain-specific setting, ConvNeXt-Tiny was first initialized with ImageNet-pretrained weights and then further pretrained on the 18-class Hi-LabSpermMorpho clinical sperm morphology dataset to obtain sperm-domain-specific pretrained weights [27]. These weights were subsequently used to initialize the SMIDS fine-tuning stage. This setting allowed the model to start from sperm morphology-oriented visual representations before being adapted to the three-class SMIDS classification task.

By comparing ImageNet initialization with Hi-LabSpermMorpho domain-specific pretraining under the same SMIDS evaluation protocol, this study aimed to determine whether sperm-domain representation learning provides a more suitable initialization for downstream sperm morphology classification. This comparison is particularly important because SMIDS is relatively small compared with large-scale natural image datasets, and a domain-aware initialization may help the model capture morphology-related structures more effectively than general-purpose visual features alone.

##### Multi-Scale Feature Extraction and Attention Refinement

Instead of relying only on the final feature representation of ConvNeXt-Tiny, the proposed framework extracts complementary feature maps from the early, middle, and late stages of the backbone. Early-stage features primarily encode low-level visual patterns, including edges, contours, and local textures; middle-stage features represent morphology-related structural cues; and late-stage features capture higher-level semantic information. This multi-scale strategy is particularly relevant to sperm morphology classification because discriminative differences may involve both subtle local irregularities and broader cell-level structures. Previous sperm morphology studies have similarly emphasized the importance of morphology-aware and structure-sensitive representation learning [21].

In the implementation, the ConvNeXt-Tiny feature extractor was divided into three sequential stage groups, denoted as [:4], [4:6], and [6:], corresponding to the early, middle, and late feature extraction stages shown in Figure 3. This grouping was not optimized based on test-set performance; instead, it was used as a fixed architectural design to exploit the hierarchical representation structure of ConvNeXt-Tiny. The early group captures low-level texture and edge information, the middle group provides intermediate morphology-related structural representations, and the late group encodes more abstract class-discriminative features. Each stage-specific output was refined using a Convolutional Block Attention Module (CBAM), which sequentially applies channel and spatial attention to emphasize discriminative morphology-related responses and suppress less informative background regions [38]. Attention-enhanced convolutional strategies have also been investigated in sperm morphology analysis, supporting the use of attention mechanisms for improving morphology-related feature representation [17].

After attention refinement, global average pooling was applied separately to the early-, middle-, and late-stage feature maps. This operation produced feature vectors with dimensions of 192, 384, and 768, respectively. The three vectors were subsequently concatenated to form a unified 1344-dimensional multi-scale representation:

(3) $$f_{\mathrm{multi}}=\left[f_{\mathrm{early}};f_{\mathrm{middle}};f_{\mathrm{late}}\right],$$

where $f_{\mathrm{early}}\in R^{192}$, $f_{\mathrm{middle}}\in R^{384}$, and $f_{\mathrm{late}}\in R^{768}$. The resulting vector $f_{\mathrm{multi}}\in R^{1344}$ was shared by the classification and supervised contrastive learning branches.

During SMIDS fine-tuning, the early stage ([:4]) was kept frozen, whereas the middle ([4:6]) and late ([6:]) stages remained trainable. This freezing strategy was applied to the ConvNeXt-Tiny model obtained from the selected initialization route: either direct ImageNet-pretrained initialization or ImageNet-pretrained weights further adapted through Hi-LabSpermMorpho domain-specific pretraining. Thus, low-level visual representations in the early stage were preserved, while the middle and late stages were allowed to adapt to SMIDS-specific morphology patterns. This partial-freezing strategy preserved low-level visual primitives learned during pretraining while allowing deeper layers to adapt to the morphology-specific structural and semantic characteristics of the SMIDS dataset. This design is consistent with the hierarchical organization of convolutional representations, in which earlier layers generally encode transferable low-level patterns and deeper layers capture increasingly task-specific information [46,47].

The unified multi-scale representation therefore combines fine-grained local morphology with higher-level structural information, providing the subsequent training branches with complementary features for distinguishing normal sperm, abnormal sperm, and non-sperm images.

#### 2.2.3. Training and Optimization

The training and optimization block consists of two sequential stages: Stage 1 supervised contrastive training and Stage 2 joint fine-tuning. During Stage 1, the original class-balanced images and their corresponding class labels are used to optimize the shared multi-scale feature extractor and projection head through the supervised contrastive objective. MixUp is not applied during this stage. After completing Stage 1, the resulting model parameters are directly used to initialize Stage 2.

During Stage 2, the shared feature extractor, projection head, and classification head are jointly optimized. The supervised contrastive branch continues to process the original balanced images and their original class labels, whereas MixUp-generated images are used exclusively in the classification branch. The resulting supervised contrastive and classification losses are combined to obtain the total Stage 2 training loss. Label smoothing and class-weighted focal loss are applied within the classification objective. The principal model, data, training, regularization, optimization, and inference settings are summarized in Table 3.

The regularization and loss-related hyperparameters listed in Table 3 were fixed before the final SMIDS evaluation. These values were selected based on literature-guided commonly used settings, training stability, and computational feasibility rather than by optimizing test-fold performance. Specifically, the SupCon temperature (τ=0.1) [37], the focal-loss focusing parameter (γ=2) [41], the MixUp parameter (α=0.1) [39], the label-smoothing coefficient (ϵ=0.05) [40], the AdamW optimizer [42], and the cosine annealing schedule with warm restarts [43] followed settings proposed or commonly used in the corresponding original studies, whereas the focal-loss class weights and the SupCon loss weight were fixed heuristically. None of these hyperparameters was tuned on the test folds; however, the held-out test fold was used for epoch-level checkpoint selection and early stopping within each training run, as described in Section 2.2.3. The sensitivity of the results to variations in the class weights, $\lambda_{sc}$, and τ was not evaluated (see Section Limitations and Future Work).

##### Stage 1: A Contrastive Branch

During Stage 1, the Contrastive Branch was trained using the original class-balanced images and their corresponding original class labels. The purpose of this stage was to organize the shared feature space before classification-oriented joint fine-tuning. MixUp was not applied, and no classification loss contributed to the Stage 1 objective. Consequently, the trainable components of the shared multi-scale feature extractor, the CBAM modules, and the projection head were optimized exclusively through supervised contrastive learning.

The unified 1344-dimensional multi-scale representation was passed to the projection head, which consisted of a fully connected layer reducing the representation from 1344 to 512 dimensions, followed by batch normalization, ReLU activation, and a final fully connected layer producing a 128-dimensional embedding. The resulting embedding was $L_{2}$-normalized before calculation of the supervised contrastive loss.

Supervised contrastive learning was used to encourage tighter same-class clustering and to increase the separability of different-class embeddings in the learned feature space [37]. Let *i* denote an anchor sample in a mini-batch, P(i) denote the set of samples sharing the same original class label as the anchor, and $z_{i}$ denote the normalized embedding of sample *i*. The supervised contrastive loss was formulated as

(4) $$L_{SupCon}=\sum_{i\in I}\frac{-1}{\left|P(i)\right|}\sum_{p\in P(i)}\log\frac{\exp(z_{i}\cdot z_{p}/\tau )}{\sum_{a\in A(i)}\exp\left(z_{i}\cdot z_{a}/\tau \right)},$$

where A(i) denotes all samples in the mini-batch except the anchor, and τ is the temperature parameter. In this study, the temperature was set to τ=0.1. Because each class-balanced mini-batch contained six samples from each of the three SMIDS classes, multiple same-class samples were available for constructing positive pairs for every anchor.

Stage 1 supervised contrastive training was performed for 20 epochs. After completion of this stage, the resulting model parameters were directly transferred to Stage 2 joint fine-tuning. Therefore, neither MixUp-generated images nor classification soft labels influenced the Stage 1 representation-learning objective.

##### Stage 2: Joint Fine-Tuning

Following Stage 1 supervised contrastive training, the model parameters obtained at the end of Stage 1 were used to initialize Stage 2 joint fine-tuning. During this stage, the trainable middle and late ConvNeXt-Tiny stages, the CBAM modules, the projection head, and the classification head were optimized jointly. The early ConvNeXt-Tiny stage remained frozen to preserve the transferable low-level visual representations learned during pretraining.

Stage 2 comprised two complementary paths sharing the same multi-scale feature extractor. In the contrastive path, the original class-balanced images and their original class labels were processed through the shared feature extractor and projection head. This path continued to optimize the supervised contrastive objective while preserving the original class relationships used to construct positive pairs.

In the B MixUp Classification Branch, the original balanced images were mixed online using MixUp with α=0.1. The resulting MixUp images were passed through the shared feature extractor and classification head, and the classification objective was calculated using class-weighted focal loss with label smoothing.

The unified 1344-dimensional multi-scale representation was passed to the classification head for three-class SMIDS prediction. The classification head first applied batch normalization, followed by a fully connected layer reducing the representation from 1344 to 512 dimensions, GELU activation, and dropout with a probability of 0.4. A second fully connected layer reduced the representation from 512 to 256 dimensions, followed by GELU activation and dropout with a probability of 0.3. The final linear layer produced three output logits corresponding to the normal sperm, abnormal sperm, and non-sperm classes.

Within each Stage 2 training iteration, the contrastive path received the original balanced images, whereas the B MixUp Classification Branch received the corresponding MixUp-generated images. The supervised contrastive loss and MixUp focal classification loss were subsequently combined to form the total Stage 2 training objective. This branch-specific separation ensured that MixUp affected only classification optimization and did not alter the original class labels used to construct positive pairs in the supervised contrastive objective.

##### B MixUp Classification Branch

During Stage 2 joint fine-tuning, MixUp was applied online exclusively within the B MixUp Classification Branch to reduce overfitting and encourage smoother classification boundaries [39]. As illustrated in Figure 3, this branch receives the original class-balanced images, generates MixUp images, and processes them through the shared multi-scale feature extractor and classification head. MixUp was not applied to the original images used by the contrastive path.

Given two images $x_{i}$ and $x_{j}$ selected from the same class-balanced mini-batch, the MixUp image was generated as

(5) $$\tilde{x}=\lambda x_{i}+\left(1-\lambda \right)x_{j},$$

where the mixing coefficient λ was sampled from a beta distribution:

(6) λ∼Beta(α,α).

In this study, the MixUp parameter was set to α=0.1. Following the implemented procedure, the sampled coefficient was adjusted as

(7) λ←max(λ,1−λ),

so that one of the paired images retained the dominant contribution to the mixed sample. The image pairing was performed by randomly permuting the samples within each class-balanced mini-batch.

The resulting MixUp images were passed through the shared feature extractor to obtain a 1344-dimensional multi-scale feature vector, which was subsequently processed by the classification head. Instead of assigning a single hard label to each mixed image, the classification loss was computed as a weighted combination of the losses associated with the two original labels:

(8) $$L_{\mathrm{MixUp}\text{-}\mathrm{cls}}=\lambda \;L_{\mathrm{cls}}\left(\tilde{x},y_{i}\right)+\left(1-\lambda \right)\;L_{\mathrm{cls}}\left(\tilde{x},y_{j}\right),$$

where $L_{\mathrm{cls}}$ denotes the class-weighted focal-loss-based classification objective incorporating label smoothing. Meanwhile, the original non-mixed images and their original labels were processed separately through the contrastive path. Therefore, MixUp affected only the B MixUp Classification Branch and did not alter the class relationships used to construct positive pairs for supervised contrastive learning.

##### Focal-Loss-Based Classification with Label Smoothing

The B MixUp Classification Branch was optimized using class-weighted focal loss incorporating label smoothing. Label smoothing was applied to reduce excessive confidence in the ground-truth class and improve generalization [40]. For a classification problem with *K* classes, the smoothed target distribution for class *k* was defined as

(9) $$y_{k}^{LS}=\left(1-\epsilon \right)y_{k}+\frac{\epsilon }{K},$$

where $y_{k}$ denotes the original one-hot target and ϵ is the label-smoothing coefficient. In this study, ϵ was set to 0.05.

Focal loss was used to reduce the contribution of easily classified samples and place greater emphasis on difficult or low-confidence examples [41]. The focal-loss-based classification objective was defined as

(10) $$L_{FL}=-\alpha_{t}{\left(1-p_{t}\right)}^{\gamma }\log\left(p_{t}\right),$$

where $\alpha_{t}$ denotes the class-dependent weighting factor, $p_{t}$ denotes the target-associated prediction probability, and γ controls the degree of emphasis placed on difficult samples. The focusing parameter was set to γ=2.

Although SMIDS exhibits only mild class imbalance, the class-dependent weights were used not only to account for differences in sample frequency but also to reflect class-dependent visual difficulty. Normal and abnormal sperm images may differ through subtle morphology-related characteristics, whereas non-sperm samples may exhibit different background and structural patterns. Therefore, the class-weighting vector was fixed before the final SMIDS evaluation as α=[1.5,1.2,1.0] for abnormal sperm, non-sperm, and normal sperm classes, respectively, and was not optimized based on test-fold performance.

##### Overall Stage 2 Training Objective

During Stage 2 joint fine-tuning, the supervised contrastive loss obtained from the original balanced images and the MixUp focal classification loss obtained from the B MixUp Classification Branch were combined as

(11) $$L_{total}=\lambda_{sc}L_{SupCon}+\left(1-\lambda_{sc}\right)L_{\mathrm{MixUp}\text{-}\mathrm{cls}},$$

where $\lambda_{sc}$ controls the contribution of the supervised contrastive objective. In this study, $\lambda_{sc}=0.5$; therefore, both branch-specific losses contributed equally to the total Stage 2 objective:

(12) $$L_{total}=0.5\;L_{SupCon}+0.5\;L_{\mathrm{MixUp}\text{-}\mathrm{cls}}.$$

During Stage 2, the model was evaluated at the end of each epoch on the held-out test fold using seven-view TTA. The checkpoint achieving the highest accuracy was retained as the final model, and early stopping with a patience of 15 epochs was applied to the same accuracy. No separate inner validation subset was used. Consequently, the reported test-fold accuracies are not independent test estimates (see Section Limitations and Future Work).

#### 2.2.4. Inference and Output

During inference, the selected Stage 2 checkpoint was used to classify the three-class SMIDS test images. As illustrated in Figure 3, each test image was first resized to 224×224 pixels and normalized, followed by seven-view test-time augmentation. The softmax probability vectors obtained from all views were averaged, and the class with the highest mean probability was selected as the final prediction.

##### Test-Time Augmentation and Final Prediction

Test-time augmentation was applied only during inference to improve prediction stability. For each test image, seven views were evaluated: the original image, horizontal flip, vertical flip, rotations of $90^{\circ }$, $180^{\circ }$, and $270^{\circ }$, and a transpose transformation.

Let *T* denote the set of test-time transformations and $p_{c}\left(T_{m}\left(x\right)\right)$ denote the softmax probability predicted for class *c* from the *m*th transformed view. The final class probability was computed as

(13) $${\bar{p}}_{c}\left(x\right)=\frac{1}{\left|T\right|}\sum_{m=1}^{\left|T\right|}p_{c}\left(T_{m}\left(x\right)\right).$$

The final prediction was obtained by selecting the class with the highest averaged probability:

(14) $$\hat{y}=\arg\max_{c}{\bar{p}}_{c}\left(x\right).$$

The resulting prediction corresponded to one of the three SMIDS classes: normal sperm, abnormal sperm, or non-sperm.

### 2.3. Ablation Study Design

To systematically evaluate the contribution of the additional training and feature-refinement components in the proposed framework, a cumulative ablation study was designed on the SMIDS benchmark dataset. The multi-scale ConvNeXt-Tiny feature extraction scheme was kept fixed across the ablation configurations, while complementary components were progressively added. Therefore, this ablation study was intended to analyze the cumulative effect of class-balanced sampling (CBS), test-time augmentation (TTA), attention-based feature refinement using CBAM, MixUp regularization, label smoothing (LS), and supervised contrastive learning (SupCon), rather than to isolate the multi-scale feature extraction strategy itself. Focal-loss-based optimization was also kept fixed across all ablation configurations and was therefore not treated as an isolated ablation factor in Table 4. The same ablation design was evaluated under ImageNet initialization and Hi-LabSpermMorpho domain-specific pretraining settings to analyze whether the observed performance changes were associated with the added components, the pretraining strategy, or their combined effect. The ablation configuration is summarized in Table 4.

Exp1, the baseline configuration, used the multi-scale ConvNeXt-Tiny classifier with the fixed class-weighted focal-loss formulation described in Section Focal-Loss-Based Classification with Label Smoothing, while label smoothing was disabled. The optimizer and learning-rate scheduler settings were kept fixed as summarized in Table 3. Exp1 did not include the additional training and feature-refinement components listed in Table 4. Exp2 introduced class-balanced sampling to reduce class dominance during optimization. Exp3 added test-time augmentation to improve inference stability. Exp4 incorporated CBAM-based attention refinement within the multi-scale feature extraction pipeline. Exp5 further introduced MixUp regularization, and Exp6 added label smoothing to reduce overconfident predictions. Finally, Exp7 combined all components, including supervised contrastive learning, to evaluate the full proposed framework.

The ablation experiments were conducted on SMIDS under different augmentation and initialization settings. Specifically, the same Exp1–Exp7 design was evaluated using two initialization routes: direct fine-tuning from ImageNet-pretrained ConvNeXt-Tiny weights and Hi-LabSpermMorpho domain-specific pretraining followed by SMIDS fine-tuning. In the Hi-LabSpermMorpho route, ConvNeXt-Tiny was first initialized with ImageNet-pretrained weights, further pretrained on Hi-LabSpermMorpho to obtain sperm-domain-specific representations, and then fine-tuned on SMIDS. The quantitative results of these experiments are reported in the Results section.

### 2.4. Evaluation Metrics

The performance of the proposed framework was evaluated using accuracy, precision, recall, and F1-score. These metrics were computed separately for each fold, and the overall performance was reported as the mean and standard deviation across the five folds.

To account for class-dependent performance differences, both macro-averaged and weighted-averaged metrics were considered. Macro averaging assigns equal importance to each class regardless of class frequency. For a dataset with *K* classes, the macro-averaged F1-score was computed as

(15) $$F1_{\mathrm{macro}}=\frac{1}{K}\sum_{k=1}^{K}F1_{k},$$

where $F1_{k}$ denotes the F1-score of class *k*. The weighted F1-score was computed as

(16) $$F1_{\mathrm{weighted}}=\sum_{k=1}^{K}\frac{n_{k}}{N}F1_{k},$$

where $n_{k}$ denotes the number of samples in class *k* and *N* denotes the total number of samples. In addition, confusion matrices were generated for the individual folds and for the combined five-fold predictions to provide a class-level interpretation of the classification performance.

### 2.5. Statistical Analysis

To support the interpretation of the ablation results, the fold-level accuracies of Exp1 (baseline) and Exp7 (full framework) were compared within each experimental setting. Because the training sets of different cross-validation folds overlap, the standard paired *t*-test underestimates the variance of the mean difference. Therefore, the corrected resampled *t*-test of Nadeau and Bengio [48] was used, in which the variance of the fold-level differences is scaled by $\left(1/k+n_{\mathrm{test}}/n_{\mathrm{train}}\right)$, with k=5 and $n_{\mathrm{test}}/n_{\mathrm{train}}=0.25$. For each setting, the mean difference and its 95% confidence interval were computed using the same corrected variance. Because the four settings reuse the same dataset and folds, they were not treated as independent replications and no pooled test was performed; the four comparisons were regarded as exploratory, and Holm-adjusted *p*-values are additionally reported to account for multiple comparisons.More generally, because the held-out test folds were also used for checkpoint selection, all model and configuration comparisons reported in this study, including the architecture comparisons, should be regarded as exploratory. The fold-level differences between Exp1 and Exp7 were also summarized descriptively across the ablation settings to support the overall interpretation of the observed performance trends. The statistical analysis was used as a supplementary interpretation of the ablation results rather than as the primary performance criterion.

## 3. Results

### 3.1. Ablation Study Results

Before conducting the cumulative ablation experiments, two training-data augmentation settings were defined to examine the robustness of the proposed framework under different levels of training-set diversity. The 1× setting refers to the original SMIDS training fold without offline expansion, whereas the 5× setting refers to a fivefold augmented training configuration in which additional augmented variants were generated only from the training images. The held-out test folds were not augmented.

The cumulative ablation study was conducted on the SMIDS dataset using two initialization routes: direct fine-tuning from ImageNet-pretrained ConvNeXt-Tiny weights and Hi-LabSpermMorpho domain-specific pretraining followed by SMIDS fine-tuning. In the Hi-LabSpermMorpho route, ConvNeXt-Tiny was first initialized with ImageNet-pretrained weights, further pretrained on Hi-LabSpermMorpho, and then fine-tuned on SMIDS. The aim was to evaluate the cumulative contribution of the proposed training and feature-refinement components across different initialization and augmentation conditions. The corresponding Exp1–Exp7 results are summarized in Table 5.

Under the 1× augmentation setting, the ImageNet-initialized model improved from 91.03% in Exp1 to 91.77% in Exp7. With Hi-LabSpermMorpho pretraining, the corresponding accuracy increased from 91.63% to 92.20%. These results indicate moderate improvements when the framework was trained using only the original SMIDS images.

Under the 5× augmentation setting, the improvements were larger. With ImageNet initialization, accuracy increased from 90.53% in Exp1 to 92.13% in Exp7. Similarly, Hi-LabSpermMorpho pretraining increased from 91.33% to 92.57%. The highest accuracy among the evaluated ablation settings was therefore achieved by Exp7 with Hi-LabSpermMorpho pretraining under 5× augmentation.

Although the performance changes were not strictly monotonic as components were progressively added, Exp7 achieved the highest accuracy under both 5× augmentation settings. Some intermediate configurations also produced competitive results, indicating that the effect of individual components depended on the initialization strategy and augmentation level.

To determine whether the improvements of Exp7 over Exp1 were statistically reliable, paired comparisons were conducted using fold-level accuracy values. As shown in Table 6, Exp7 achieved higher mean accuracy than Exp1 in each of the four evaluated conditions: 1× ImageNet, 1× Hi-LabSpermMorpho, 5× ImageNet, and 5× Hi-LabSpermMorpho. The improvements under the 1× settings were not statistically significant, whereas significant improvements were observed under both 5× settings.

When the fold-level differences from all four evaluated conditions were summarized descriptively, Exp7 showed an average improvement of 1.04 percentage points over Exp1. This value was used only to describe the overall trend across ablation settings and was not treated as an independent primary statistical test. Overall, these findings show that the full Exp7 configuration provided the clearest and statistically significant gains under the 5× augmentation settings.

### 3.2. Design Alternatives and Component Removal

To examine individual design choices, targeted experiments were conducted in which a single element of the full framework was modified or removed while all other settings were kept unchanged. All variants used Hi-LabSpermMorpho pretraining, 5× augmentation, the same folds, the same training schedule, and the same checkpoint-selection rule; therefore, the training budget was identical across variants (Table 7).

The targeted experiments did not show a statistically significant advantage for any individual design alternative over the full framework (p≥0.35 for all comparisons). Although some alternatives achieved slightly higher mean accuracy, the full framework maintained one of the lowest fold-to-fold variabilities (SD = 0.35) and a comparatively high worst-fold accuracy (92.00%). These descriptive differences in fold-to-fold variability were not formally tested and should not be interpreted as evidence of superior stability. Removing class-balanced sampling or MixUp increased the variability (SD = 0.87 and 0.85) and reduced the worst-fold accuracy (91.17% and 90.83%). Regarding the training budget, the framework includes a 20-epoch supervised contrastive stage (Stage 1) before joint fine-tuning (Stage 2), intended to support the learning of morphology-sensitive and class-discriminative representations before the final classification objective is optimized. This two-stage design is based on the protocol of the original supervised contrastive learning study, in which contrastive representation learning precedes classification-oriented training [37]. Because all variants in Table 7 followed the same two-stage schedule, the component-removal comparisons were not affected by training-budget differences. In addition, offline augmentation increases the number of optimization steps per epoch (approximately 130, 650, and 1300 iterations for 1×, 5×, and 10×), and comparisons between initialization strategies were always performed under identical budgets. These targeted experiments also help interpret the non-monotonic steps observed in the cumulative ablation. Because each component was removed from the full framework while the remaining components were retained, the analysis provides a conditional assessment of the contribution of each component within the complete framework. No individual modification changed the mean accuracy by more than 0.17 percentage points, which was smaller than the fold-to-fold variability observed for most variants. This suggests that the non-monotonic behavior may reflect partially overlapping mechanisms rather than large independent effects of individual components. For example, MixUp and label smoothing both regularize the classification target, whereas class-balanced sampling and class-weighted focal loss both influence the treatment of class imbalance and difficult samples. Consequently, the marginal effect of one component may diminish when a related mechanism is already present. These observations should not, however, be interpreted as a quantitative isolation of specific pairwise interaction effects.

### 3.3. Effect of Domain-Specific Pretraining Under the Full Framework

Following the cumulative ablation analysis, the effect of domain-specific pretraining was evaluated using Exp7, which represents the complete proposed framework. The cumulative ablation study was limited to the 1× and 5× augmentation settings to evaluate the progressive contribution of the proposed components. After this analysis, the complete Exp7 configuration was additionally evaluated under a 10× augmentation setting to examine whether the effect of Hi-LabSpermMorpho domain-specific pretraining remained consistent when the training-data diversity was further increased. Therefore, the 10× setting was used only in the final initialization comparison and was not included in the cumulative ablation table.

Hi-LabSpermMorpho domain-specific pretraining was then compared with direct ImageNet-pretrained initialization on the SMIDS dataset under the 1×, 5×, and 10× augmentation settings. In the Hi-LabSpermMorpho route, ConvNeXt-Tiny was first initialized with ImageNet-pretrained weights, further pretrained on Hi-LabSpermMorpho, and then fine-tuned on SMIDS. As summarized in Table 8, the highest performance obtained with the complete Exp7 configuration was achieved using Hi-LabSpermMorpho pretraining and 10× augmented training data, with an average accuracy of 92.73%.

In addition to the fold-level accuracy results, macro-averaged precision, recall, and F1-score were calculated from the combined five-fold confusion matrices to provide a more balanced evaluation across the three SMIDS classes.

As shown in Table 9, Hi-LabSpermMorpho domain-specific pretraining achieved higher macro F1-scores than direct ImageNet initialization under all augmentation settings. The best overall result was obtained with Hi-LabSpermMorpho pretraining and 10× augmentation, achieving 92.73% accuracy and 92.79% macro F1-score. These findings show that the improvement obtained with domain-specific pretraining was not limited to accuracy, but was also reflected in macro-averaged precision, recall, and F1-score.

**Figure 4:**
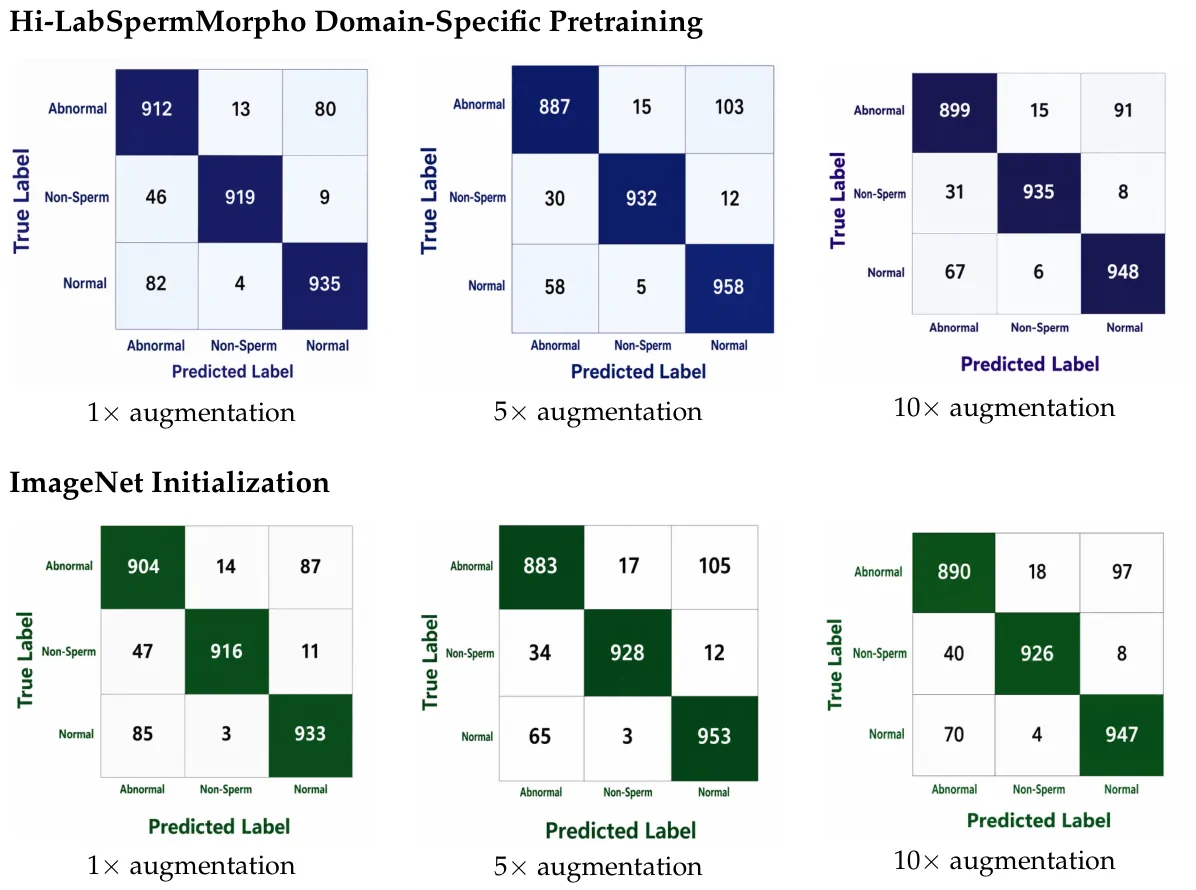
Confusion matrices for SMIDS classification under Hi-LabSpermMorpho domain-specific pretraining (blue) and ImageNet initialization (green) across 1×, 5×, and 10× augmentation settings.

The class-level performance of the complete Exp7 framework was further examined using the combined five-fold confusion matrices shown in Figure 4. The Hi-LabSpermMorpho-pretrained models exhibited strong diagonal dominance across the three augmentation settings, indicating that most samples were assigned to their corresponding SMIDS classes. The Hi-LabSpermMorpho configuration with 10× augmentation, which achieved the highest average accuracy of 92.73%, also maintained consistent classification performance across the abnormal sperm, non-sperm, and normal sperm classes. Although similar patterns were observed with ImageNet initialization, Hi-LabSpermMorpho pretraining achieved higher average accuracy under all three augmentation settings.

### 3.4. Comparison with Previous Benchmark Results

**Figure 5:**
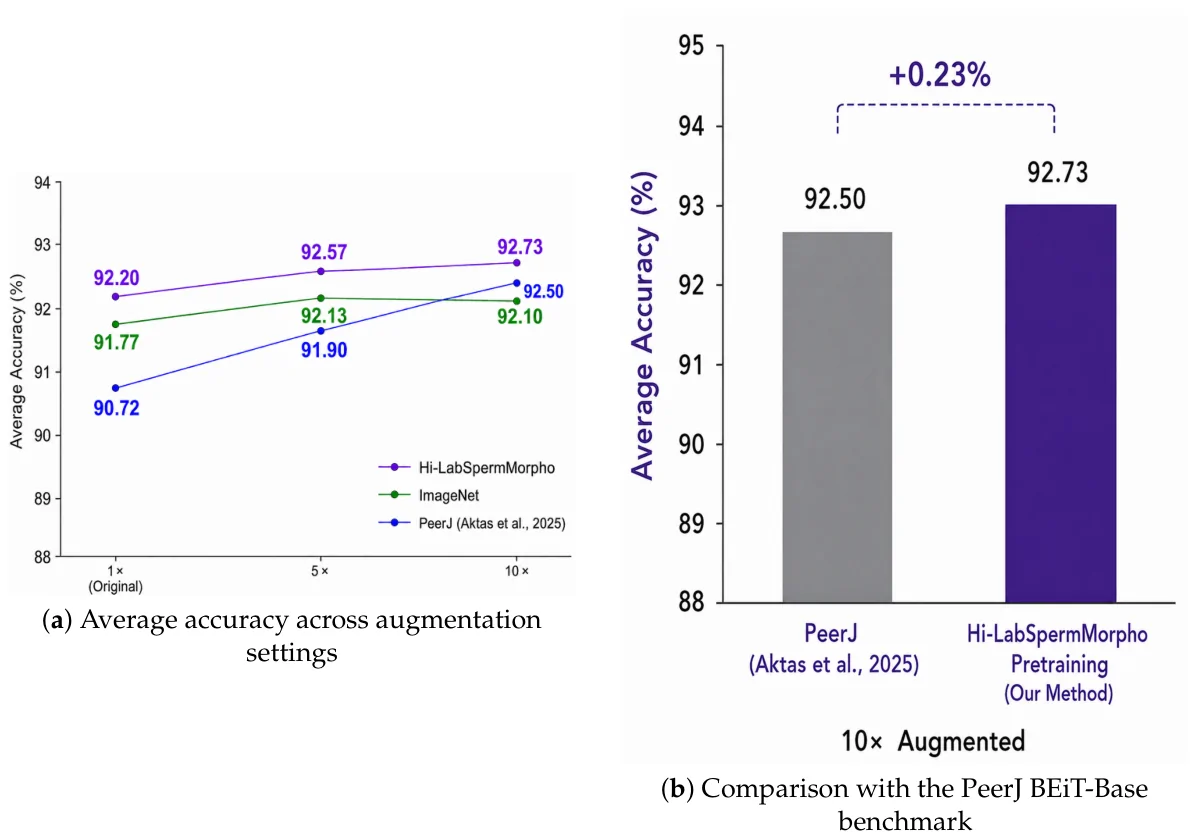
Performance comparison on the SMIDS dataset. (**a**) Average classification accuracy across the evaluated training-data augmentation settings for Hi-LabSpermMorpho domain-specific pretraining, direct ImageNet-pretrained initialization, and the previously reported PeerJ BEiT-Base benchmark [20]. (**b**) Benchmark comparison between the best proposed configuration and the previously reported PeerJ BEiT-Base result. The 10× augmentation setting was used only as an additional final comparison for the complete Exp7 configuration to examine the effect of increased training-data diversity, rather than as part of the cumulative ablation analysis. For the proposed framework, values are reported as the mean accuracy across the five folds ± fold-to-fold standard deviation: Hi-LabSpermMorpho pretraining, 92.20 ± 0.84% (1×), 92.57 ± 0.35% (5×), and 92.73 ± 0.43% (10×); ImageNet initialization, 91.77 ± 0.74% (1×), 92.13 ± 0.62% (5×), and 92.10 ± 0.79% (10×). The PeerJ BEiT-Base values are shown as reported in the original publication; fold-level dispersion was not available for these previously published results.

To further compare the proposed framework with the previously reported SMIDS benchmark, the average accuracy values obtained using the full Exp7 configuration were visualized across different augmentation settings. As shown in Figure 5, the proposed Exp7 framework with Hi-LabSpermMorpho pretraining yielded numerically higher average accuracy than the same Exp7 framework with ImageNet initialization and the reported PeerJ BEiT-Base benchmark across the evaluated augmentation levels.

Under the 1× setting, the full Exp7 framework achieved 92.20% accuracy with Hi-LabSpermMorpho pretraining, compared with 91.77% using ImageNet initialization and 90.72% for the PeerJ benchmark. Under the 10× augmentation setting, the proposed Exp7 framework with Hi-LabSpermMorpho pretraining achieved its highest average accuracy of 92.73%, which was numerically higher than the previously reported PeerJ BEiT-Base benchmark accuracy of 92.50%. Although the 10× augmentation setting provided the highest average accuracy in the final comparison, it also increases the training-data volume and computational cost. Therefore, the 10× setting should be interpreted as an additional robustness-oriented evaluation of the complete framework rather than as the main ablation setting. The 1× and 5× settings were used for the cumulative ablation analysis, whereas the 10× setting was included only to examine whether the domain-specific pretraining effect remained stable under increased training-data diversity.

These results indicate that domain-specific pretraining provides a more effective initialization for SMIDS classification than ImageNet initialization and allows the ConvNeXt-Tiny-based framework to achieve competitive performance relative to the transformer-based PeerJ benchmark.

### 3.5. Comparison with Alternative Architectures Under Identical Conditions

To provide a more direct and controlled comparison, EfficientNetV2-S and Swin-T were trained and evaluated using the same five SMIDS folds, the same 5× augmented training data, the same seven-view test-time augmentation, and the same model-selection procedure used for the proposed framework. This comparison was designed to reduce the methodological differences inherent in comparisons with previously published benchmark results.

As shown in Table 10, under these matched experimental conditions, the Hi-LabSpermMorpho-pretrained proposed framework achieved the highest mean accuracy (92.57%) and the lowest fold-to-fold variability (SD = 0.35). It achieved higher accuracy than EfficientNetV2-S and Swin-T in four of the five folds. In contrast, when the proposed framework was initialized only with ImageNet weights, its mean accuracy (92.13%) was identical to that of EfficientNetV2-S. These findings indicate that the performance advantage of the proposed framework is primarily associated with sperm-domain-specific pretraining, rather than with the backbone architecture alone. Using the corrected resampled *t*-test, which accounts for the dependence between cross-validation folds, these differences did not reach statistical significance with five folds (p=0.25 versus EfficientNetV2-S and p=0.20 versus Swin-T). In particular, the 0.23 percentage-point difference between the proposed framework (92.73%) and the previously reported BEiT-Base benchmark (92.50%) should not be interpreted as direct evidence of superiority, because these results were obtained in separate studies under different experimental settings. To further examine whether the observed performance is specific to the convolutional backbone, the ConvNeXt-Tiny backbone of the proposed framework was replaced with BEiT-Base, while all other components, folds, augmentation settings, and the evaluation protocol were kept unchanged. For the domain-specific setting, BEiT-Base was pretrained on Hi-LabSpermMorpho using the same procedure as ConvNeXt-Tiny. The results are presented in Table 11.

**Table 10:** Five-fold classification accuracy (%) of different architectures evaluated under identical SMIDS experimental conditions.

Model	Initialization	Fold 1	Fold 2	Fold 3	Fold 4	Fold 5	Mean ± SD
Proposed framework	Hi-LabSpermMorpho	92.32	92.85	92.83	92.00	92.83	92.57 ± 0.35
Proposed framework	ImageNet	92.82	92.18	92.50	91.00	92.17	92.13 ± 0.62
EfficientNetV2-S	ImageNet	91.82	92.51	92.00	91.17	93.17	92.13 ± 0.67
Swin-T	ImageNet	92.65	90.68	92.33	90.33	91.83	91.57 ± 0.91

**Table 11:** Five-fold SMIDS accuracy (%) of the proposed framework with ConvNeXt-Tiny and BEiT-Base backbones under identical conditions. Total parameters within the full framework: ConvNeXt-Tiny 29.5 M; BEiT-Base 88.5 M (85.8 M for the backbone with a classification head, Table 12).

Initialization	Backbone	Aug.	Fold 1	Fold 2	Fold 3	Fold 4	Fold 5	Mean ± SD
ImageNet	ConvNeXt-Tiny	1×	91.65	91.35	92.67	90.67	92.50	91.77 ± 0.74
ImageNet	BEiT-Base	1×	91.15	90.85	93.00	90.00	91.83	91.37 ± 1.01
ImageNet	ConvNeXt-Tiny	5×	92.82	92.18	92.50	91.00	92.17	92.13 ± 0.62
ImageNet	BEiT-Base	5×	91.32	92.85	92.67	89.67	92.00	91.70 ± 1.15
ImageNet	ConvNeXt-Tiny	10×	92.49	92.68	92.83	90.67	91.83	92.10 ± 0.79
ImageNet	BEiT-Base	10×	91.49	92.18	91.83	89.83	91.17	91.30 ± 0.81
Hi-LabSpermMorpho	ConvNeXt-Tiny	1×	92.32	93.18	92.67	90.67	92.17	92.20 ± 0.84
Hi-LabSpermMorpho	BEiT-Base	1×	91.32	93.01	94.33	90.00	92.67	92.27 ± 1.48
Hi-LabSpermMorpho	ConvNeXt-Tiny	5×	92.32	92.85	92.83	92.00	92.83	92.57 ± 0.35
Hi-LabSpermMorpho	BEiT-Base	5×	91.49	92.68	93.67	90.17	92.17	92.04 ± 1.17
Hi-LabSpermMorpho	ConvNeXt-Tiny	10×	92.82	93.34	92.33	92.17	93.00	92.73 ± 0.43
Hi-LabSpermMorpho	BEiT-Base	10×	91.49	93.01	94.00	89.50	92.50	92.10 ± 1.53

**Table 12:** Computational cost of the compared architectures using a single 224×224 input view. For BEiT-Base, the backbone with a classification head was measured.

Model	Parameters (M)	GFLOPs/Image	Time/Image, bs = 1 (ms)	Time/Image, bs = 18 (ms)
Proposed framework	29.5	8.91	5.52	0.59
Swin-T	28.1	8.99	9.43	0.66
EfficientNetV2-S	21.0	5.70	13.08	0.70
BEiT-Base	85.8	35.13	6.61	1.88

With the same ImageNet initialization, ConvNeXt-Tiny achieved a higher mean accuracy than BEiT-Base under all three augmentation settings (+0.40, +0.43, and +0.80 percentage points for 1×, 5×, and 10×, respectively). The difference was statistically significant only under the 10× setting, in which ConvNeXt-Tiny achieved higher accuracy in all five folds (92.10% vs. 91.30%; p=0.005, corrected resampled *t*-test), whereas the differences under the 1× and 5× settings were not significant (p=0.23 and p=0.53, respectively). These results were obtained with approximately three times fewer parameters for ConvNeXt-Tiny. Hi-LabSpermMorpho pretraining also improved the performance of BEiT-Base under all three settings, indicating that the benefit of sperm-domain pretraining generalizes across architectures. With domain-specific pretraining, the highest overall accuracy (92.73%) and consistently lower fold-to-fold variability (SD = 0.35–0.84 vs. 1.17–1.53) were obtained with ConvNeXt-Tiny, although the differences between the two pretrained backbones were not statistically significant.

### 3.6. Computational Cost

To assess the practical cost of the proposed framework, the number of parameters, floating-point operations (FLOPs), and per-image inference time were measured for all compared architectures using the same procedure and hardware (a single NVIDIA GeForce RTX 5090 GPU).FLOPs were counted with PyTorch 2.6.0 profiler, which also accounts for the matrix multiplications in transformer attention layers. Inference time was measured after warm-up for batch sizes of 1 and 18. The results are summarized in Table 12.

Inference time is influenced not only by parameter count and FLOPs but also by architectural structure and hardware-specific execution efficiency. Therefore, larger models may occasionally show lower latency than smaller models under identical measurement conditions.

The proposed framework achieved the lowest inference time among all compared architectures for both single-image and batched inference. Compared with BEiT-Base, it requires approximately three times fewer parameters and four times fewer FLOPs, while also providing approximately threefold faster batched inference and higher classification accuracy under matched experimental conditions (Table 11). The training-specific components, including class-balanced sampling, MixUp, label smoothing, and focal-loss-based optimization, are used only during training and therefore introduce no additional inference cost. Likewise, Hi-LabSpermMorpho pretraining is performed only once before SMIDS fine-tuning and does not affect deployment-time complexity. Seven-view TTA increases inference cost approximately sevenfold, from 5.52 to 38.30 ms per image for batch size 1 and from 0.59 to 4.21 ms per image for batch size 18. However, for the Hi-LabSpermMorpho-pretrained model with 10× augmentation, TTA improved accuracy from 91.07% to 92.73%. Thus, TTA represents an explicit accuracy–efficiency trade-off and can be disabled when inference speed is prioritized. Regarding the biological validity of the TTA transformations, spermatozoa in stained smears may appear in arbitrary orientations, while morphological assessment is primarily based on the shape and dimensions of the head, midpiece, and tail rather than on the global orientation of the cell in the image. Rotations by multiples of 90°, flips, and transposition are geometry-preserving transformations and therefore retain the principal morphological structures used for classification. To examine empirically whether these transformations adversely affect performance, their contribution was analyzed for the best configuration (Hi-LabSpermMorpho pretraining, 10× augmentation). Individual views achieved 90.73–91.73% accuracy, with 91.07% for the original view. Combining the original view with flips increased the accuracy to 91.83%, adding the rotation-based views increased it to 92.43%, and using all seven views yielded the highest accuracy of 92.73%. These results indicate that, in the present dataset, the combined orientation-based TTA strategy provides a net performance benefit rather than degrading classification performance.

### 3.7. Qualitative Error Analysis

**Figure 6:**
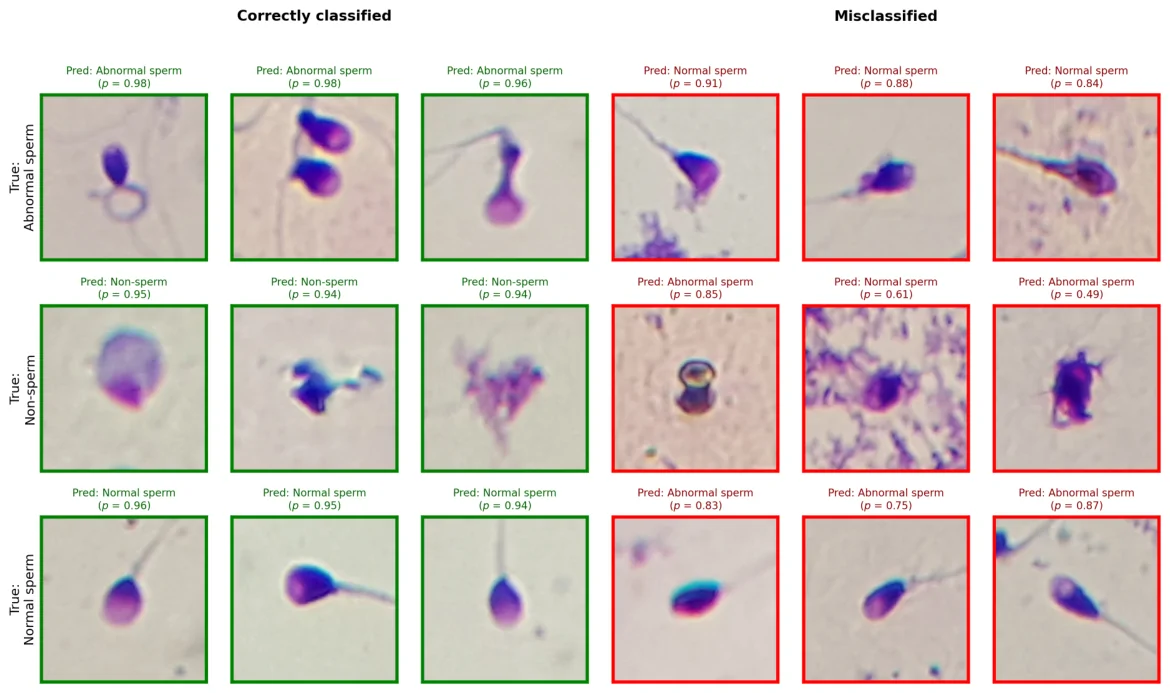
Representative correctly classified (green frames) and misclassified (red frames) SMIDS test images for the proposed framework with Hi-LabSpermMorpho pretraining and 10× augmentation. Rows indicate the true class; the predicted class and its probability (averaged over seven-view TTA) are shown above each image.

To complement the confusion matrices, representative correctly classified and misclassified test images of the best-performing configuration (Hi-LabSpermMorpho pretraining, 10× augmentation) are shown in Figure 6. Each image was evaluated by the model trained on the fold in which it served as a test sample. For each class, three correctly classified and three misclassified images, selected by the authors as representative of the typical predictions and error patterns, are shown.

Across the 3000 out-of-fold test predictions, 218 samples were misclassified. Most errors (158 of 218) occurred between the normal and abnormal sperm classes, including 91 abnormal sperm samples predicted as normal and 67 normal sperm samples predicted as abnormal, whereas confusion involving the non-sperm class was less frequent (60 of 218). Consistently, class-wise recall was highest for non-sperm (96.0%), followed by normal sperm (92.9%) and abnormal sperm (89.5%). Visual inspection of the misclassified samples in Figure 6 shows that several abnormal sperm predicted as normal exhibit head-shape irregularities that are partly obscured by adjacent debris or background structures. Conversely, some normal sperm predicted as abnormal show regular oval heads with only minor contour variations. Non-sperm samples predicted as sperm include rounded or compact debris resembling sperm heads.These examples indicate that the remaining classification errors are concentrated primarily in visually ambiguous samples, particularly at the normal–abnormal class boundary. Class-specific performance with 95% confidence intervals is reported in Table 13. The balanced accuracy (92.77%) was nearly identical to the overall accuracy (92.73%), indicating that the performance was not driven by a single class. The lowest recall was observed for abnormal sperm (89.45%), mainly because of the 91 abnormal samples classified as normal, which largely lie near the normal–abnormal boundary.

### 3.8. Grad-CAM Visualization

**Figure 7:**
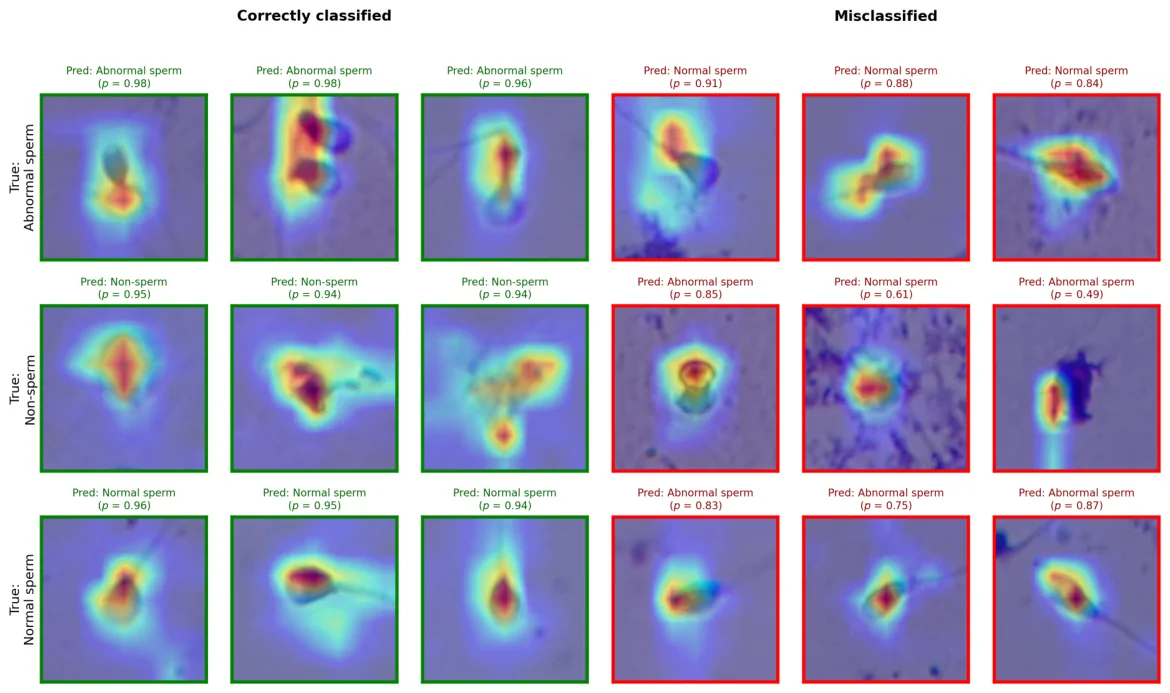
Grad-CAM visualizations for representative correctly classified (green frames) and misclassified (red frames) SMIDS test samples. Rows indicate the ground-truth class, while the predicted class and its probability are shown above each image. Warmer regions indicate areas with stronger contribution to the model prediction.

To further examine the image regions contributing to the model predictions, Grad-CAM visualizations [49] were generated for representative correctly classified and misclassified SMIDS test samples from the best-performing configuration (ConvNeXt-Tiny with Hi-LabSpermMorpho pretraining and 10× augmentation). Grad-CAM was applied to the CBAM-refined output of the late ConvNeXt-Tiny stage with respect to the predicted class, using the model of the fold in which each image served as a test sample. The resulting activation maps were overlaid on the original images, with warmer regions indicating image areas that contributed more strongly to the predicted class. Because the maps are derived from a 7×7 feature map, they provide a coarse localization. Representative examples are shown in Figure 7. The images and their arrangement are identical to those in Figure 6, allowing each activation map to be compared directly with the corresponding original image and its classification result.

The Grad-CAM maps show that, for correctly classified sperm samples, the strongest activations are generally concentrated on the sperm-associated structures rather than on the surrounding background. For abnormal and normal sperm, prominent responses are frequently observed around the head and adjacent morphological structures, whereas non-sperm predictions are driven by the visually distinctive structures present in the corresponding patches. In the misclassified examples, the activations were less consistently confined to the cell and partly extended to adjacent structures or background regions, consistent with the qualitative analysis. These visualizations provide complementary evidence regarding the image regions used by the model when forming its predictions, although they should be interpreted as post-hoc explanations rather than direct evidence of clinical decision-making.

## 4. Discussion

This study proposes a domain-specific ConvNeXt-Tiny-based framework for sperm morphology classification on the SMIDS benchmark dataset. The framework was designed to address several challenges commonly encountered in biomedical image classification, including limited dataset size, subtle inter-class visual differences, potential class imbalance, and sensitivity to augmentation strategies. Unlike studies that primarily compare different backbone architectures, the present study focuses on the combined effect of domain-specific pretraining, multi-scale attention-guided feature extraction, and robust training components within a ConvNeXt-Tiny-based convolutional framework.

The use of Hi-LabSpermMorpho pretraining provides a more task-relevant initialization than general-purpose ImageNet initialization because the model is exposed to sperm morphology-related visual structures before being fine-tuned on SMIDS. This is particularly important because SMIDS is relatively small compared with large-scale natural image datasets and contains visually related categories such as normal sperm, abnormal sperm, and non-sperm. Previous sperm morphology studies have shown that feature fusion, ensemble learning, and deep representation learning can improve classification performance under limited-data conditions [14,15]. In this context, the observed improvement obtained with Hi-LabSpermMorpho pretraining, especially under the 10× augmentation setting, supports the importance of sperm-domain representation learning for SMIDS classification.

The multi-scale feature extraction strategy was included as an architectural design component of the proposed framework and was additionally examined in Section 3.2. Sperm morphology classification may depend on both fine-grained local details and broader structural patterns. Early-stage ConvNeXt features can capture local edge, contour, and texture information, whereas deeper features encode more abstract morphology-related representations. This interpretation is consistent with previous studies emphasizing the importance of morphology-aware feature extraction and anatomical representation learning in sperm image analysis [16,21]. By combining early, middle, and late features into a unified representation, the proposed framework was designed to preserve complementary information across multiple visual scales. The attention-based refinement modules were then applied to emphasize discriminative regions while reducing the influence of less informative background responses.

The ablation study provides a systematic way to interpret the cumulative contribution of the additional training and feature-refinement components evaluated in this study. The SMIDS ablation results showed that the effect of these components was not strictly monotonic across all configurations, indicating that the final performance depends on the interaction between initialization strategy, augmentation level, and training components. This observation is compatible with recent sperm morphology studies showing that performance improvements may arise from combinations of model fusion, attention mechanisms, knowledge distillation, or ensemble-based learning rather than from a single isolated component [22,50]. Under the 1× setting, Hi-LabSpermMorpho pretraining provided stronger performance than ImageNet initialization in the final Exp7 configuration. Under the 5× setting, the full Exp7 configuration achieved the best performance for both ImageNet initialization and Hi-LabSpermMorpho pretraining, with the highest ablation result obtained using Hi-LabSpermMorpho pretraining. These findings indicate that the complete Exp7 configuration performed best in the cumulative ablation under the 5× setting; however, the targeted component-removal experiments in Section 3.2 do not establish that each individual component is necessary.The paired statistical analysis further supports this interpretation: although the 1× settings showed only modest and non-significant improvements, the full Exp7 configuration produced statistically significant gains under the 5× augmentation settings and positive mean differences in all four evaluated settings. These gains indicate a benefit of the combined training strategy relative to the baseline configuration. However, because the targeted component-removal experiments (Table 7) showed similar performance across several configurations and no statistically significant advantage for the full framework, these results do not establish the necessity or superiority of the complete combination of components.

Compared with previous benchmark studies, the proposed framework is positioned as a ConvNeXt-Tiny-based convolutional alternative enhanced by domain-specific representation learning and training-level improvements. Recent studies have explored transformer-based, ensemble-based, and category-aware strategies for sperm morphology classification, demonstrating that different model families can provide complementary advantages depending on dataset size, feature representation, and training design [19,23]. In particular, the comparison with the BEiT-Base benchmark reported by Aktas et al. is important because that study demonstrated strong transformer-based performance on SMIDS, achieving 92.50% accuracy [20]. In the present study, the best SMIDS result reached 92.73% with Hi-LabSpermMorpho pretraining and 10× augmentation. Although this result is numerically higher than the reported BEiT-Base benchmark, the improvement is modest and should be interpreted as competitive performance rather than evidence of statistically significant superiority. A more recent study by Kılıç reported a higher SMIDS accuracy of 96.08% using a CBAM-enhanced ResNet50 combined with a deep feature engineering pipeline [17]. However, this result should be interpreted with caution when compared directly with the present study. First, the reported best-performing SMIDS configuration was not a purely end-to-end CNN classifier, but a hybrid pipeline in which GAP features extracted from a ResNet50+CBAM model were reduced using PCA and classified using an SVM with an RBF kernel. Second, the evaluation protocol is not described in a fully consistent manner: the abstract reports evaluation using 5-fold cross-validation, whereas the implementation details describe a 70% training, 15% validation, and 15% testing partition. This difference in the reported partitioning makes it difficult to determine how model selection, feature selection, and final testing were organized, and therefore limits the direct comparability of the reported accuracy with the cross-validation results of the present study, in which all compared architectures were evaluated on identical folds (Section 3.5). In addition, the reported SMIDS class-wise results and confusion matrix appear to correspond to a limited test subset rather than the complete 3000-image dataset. Therefore, although Kılıç reported a higher numerical SMIDS accuracy, the result is based on a hybrid deep feature engineering and classical machine learning pipeline with a different and partially ambiguous evaluation protocol. For this reason, the present study does not claim state-of-the-art performance based solely on the highest reported accuracy; instead, it positions the proposed method as a ConvNeXt-Tiny-based end-to-end deep learning framework evaluated under a clearly defined cross-validation and ablation protocol.

Overall, the findings indicate that the contribution of the proposed framework is not limited to the final accuracy value alone. Rather, the study provides a structured evaluation of how domain-specific pretraining, multi-scale feature extraction, attention refinement, balanced sampling, test-time augmentation, and regularization strategies can be integrated into a ConvNeXt-Tiny-based convolutional model for SMIDS-based sperm morphology classification. This is particularly relevant for biomedical image classification tasks, where dataset size is often limited and performance differences should be interpreted together with methodological robustness and component-level analysis. Recent multi-level ensemble, sequential deep learning, and morphology-oriented learning studies also support the importance of robust model design for sperm morphology assessment, especially when working with limited benchmark datasets [18,51,52].

### Limitations and Future Work

This study has several limitations. First, although SMIDS is a widely used benchmark dataset for sperm morphology classification, its size remains limited compared with large-scale medical imaging datasets. Therefore, the reported results should be interpreted within the scope of this benchmark dataset. In addition, because subject identifiers are not available in the public SMIDS release, the cross-validation was performed at the patch level, and patches from the same subject or microscopic field may appear in different folds; therefore, the results reflect patch-level benchmark performance and should not be interpreted as evidence of generalization to unseen subjects; group-wise validation on datasets with traceable subject identifiers is required for this purpose.Furthermore, no separate inner validation subset was used; the held-out test fold was used for epoch-level checkpoint selection and early stopping. This introduces selection bias; therefore, the reported accuracies cannot be regarded as independent test estimates and may be optimistic. Comparisons between models and configurations should accordingly be regarded as exploratory. Independent validation, such as nested cross-validation with an inner validation set or evaluation on an external dataset, is required to establish the reported performance gains. In addition, the focal-loss class weights, the SupCon loss weight ($\lambda_{sc}$), and the temperature parameter (τ) were fixed before the final evaluation, and the sensitivity of the results to reasonable variations in these hyperparameters was not examined. Therefore, the reported performance may depend on these specific settings, and a systematic sensitivity analysis should be considered in future work. Second, although Grad-CAM visualizations were incorporated to provide preliminary insight into image regions contributing to model predictions, such post-hoc interpretability methods should be considered complementary rather than definitive explanations of model behavior [53]. These visualizations do not provide direct anatomical localization or segmentation of sperm structures and should not be interpreted as a substitute for clinically validated region-level annotation. Third, the generalization ability of the proposed model across different laboratories, imaging devices, staining protocols, and clinical populations requires further validation. This is important because medical image classification models may experience performance degradation when applied to data acquired from different domains or clinical settings [54,55]. Although Hi-LabSpermMorpho pretraining was used to incorporate domain-specific sperm morphology information, additional external validation datasets would strengthen the clinical applicability of the proposed approach.Moreover, no independent clinician assessment or prospective clinical validation was performed; therefore, the interpretability analyses should be regarded as preliminary. It should also be emphasized that all reported results refer to patch-level classification on the SMIDS benchmark and should not be equated with clinical sperm morphology assessment, and that the use of Hi-LabSpermMorpho for pretraining does not constitute external validation.

Future work will focus on evaluating the proposed framework on larger multi-center sperm morphology datasets, incorporating external validation cohorts, and extending the current interpretability analysis through expert-guided assessment and more detailed region-level explanations. In particular, future studies will investigate whether the regions highlighted by Grad-CAM correspond consistently to clinically relevant morphological structures as assessed by domain experts. In addition, integrating segmentation-aware modules or multi-task learning strategies may further improve the ability of the model to explicitly localize clinically relevant sperm structures. This direction is also supported by recent sperm analysis studies that emphasize the importance of segmentation-oriented preprocessing and structure-aware analysis for morphology-related tasks [56].

## 5. Conclusions

This study presented a domain-specific ConvNeXt-Tiny-based framework for automated sperm morphology classification on the SMIDS benchmark dataset. The proposed approach integrates Hi-LabSpermMorpho-based domain-specific pretraining, multi-scale feature extraction, attention-guided feature refinement, class-balanced sampling, test-time augmentation, MixUp, label smoothing, focal-loss-based optimization, and supervised contrastive learning. By combining these components, the framework was designed to improve classification robustness under limited-data conditions and to better capture morphology-related visual patterns in sperm images.

A key aspect of the proposed framework is the use of sperm-domain pretraining. Instead of relying only on general-purpose ImageNet initialization, the model was initialized using representations learned from the Hi-LabSpermMorpho dataset, which contains clinically derived sperm morphology images from the Hi-LabSpermMorpho data source. This strategy provided a task-relevant starting point for downstream fine-tuning on SMIDS. In addition, the multi-scale feature extraction design allowed the model to combine low-level texture information, mid-level structural patterns, and high-level semantic features within a unified representation.

The experimental results showed that Hi-LabSpermMorpho domain-specific pretraining improved SMIDS classification performance compared with ImageNet initialization. The best performance was achieved with Hi-LabSpermMorpho pretraining and 10× augmented SMIDS data, reaching 92.73% average patch-level accuracy. Under matched experimental conditions, the proposed framework achieved the numerically highest mean accuracy among the evaluated architectures; however, the differences between the proposed framework and EfficientNetV2-S, Swin-T, and domain-pretrained BEiT-Base were not statistically significant. The only significant backbone difference was observed in the ImageNet-initialized 10× comparison with BEiT-Base. The 0.23 percentage-point difference from the previously reported BEiT-Base result (92.50%) [20] should be interpreted only as a contextual cross-study comparison.

The ablation study clarified the cumulative contribution of the evaluated training and feature-refinement components under different SMIDS augmentation and initialization settings. The results showed that performance changes were not strictly monotonic across all configurations, suggesting that the effectiveness of individual components depends on their interaction with augmentation level and initialization strategy. The full Exp7 configuration achieved significant gains over the baseline configuration under the 5× settings; however, the targeted component-removal experiments showed similar performance across several configurations and did not establish the necessity or superiority of the complete combination of components.

Overall, the findings suggest that domain-specific pretraining and carefully designed training strategies can strengthen ConvNeXt-Tiny-based convolutional architectures for sperm morphology classification. Independent validation is required to confirm the reported performance gains. Future work should further validate the proposed approach on larger multi-center datasets, investigate external clinical generalization, and incorporate interpretability analyses to better understand the morphology-related regions used by the model during SMIDS classification.

## Figures and Tables

**Table 1:** Summary of the datasets employed in this study.

Dataset	Role	Number of Images	Classes
SMIDS	Target benchmark dataset	3000	Normal sperm Abnormal sperm Non-sperm
Hi-LabSpermMorpho	Domain-specific pretraining source	49.345	18 Sperm Morphology Classes

**Table 2:** Morphological classes and number of images in Hi-LabSpermMorpho used for domain-specific pretraining.

Region	Class	Images
Head	AmorfHead	7968
	NarrowAcrosome	5401
	PyriformHead	3943
	TaperedHead	3905
	VacuolatedHead	2810
	PinHead	1322
	RoundHead	873
	DoubleHead	135
Neck/midpiece	ThickNeck	4251
	TwistedNeck	3574
	AsymmetricNeck	1316
	ThinNeck	545
Tail	TwistedTail	5481
	CurlyTail	3782
	ShortTail	1888
	DoubleTail	464
	LongTail	276
Normal	Normal	1411
	Total	49,345

**Table 3:** Main model, data, training, optimization, and inference settings used in the proposed framework.

Category	Component	Setting
Model	Backbone	ConvNeXt-Tiny
	Input image size	224 × 224 pixels
	Initialization strategies	ImageNet-pretrained weights (general-domain initialization) or Hi-LabSpermMorpho-pretrained weights (sperm-domain initialization)
Data	Target dataset	SMIDS
	Cross-validation	5-fold cross-validation
	Balanced batch	6 samples per class
	Batch size	18
Training	Stage 1	Supervised contrastive training for 20 epochs
	Stage 2	Joint fine-tuning for up to 40 epochs
Regularization	MixUp	α=0.1
	Label smoothing	ϵ=0.05
	Focal loss	γ=2, α=[1.5,1.2,1.0] for abnormal sperm, non-sperm, and normal sperm classes
	Focal loss with label smoothing	Focal modulation applied to the sample-wise label-smoothed cross-entropy $\ell_{i}$, with $p_{t}=\exp\left(-\ell_{i}\right)$, followed by mean reduction over the batch
	SupCon temperature	τ=0.1
	SupCon loss weight	$\lambda_{sc}=0.5$
	SupCon reduction	Mean over positive samples per anchor, followed by mean over anchors with at least one positive sample
	Stage 2 total loss	$0.5\;L_{\mathrm{SupCon}}$ (original images) $+\;0.5\;L_{\mathrm{cls}}$ (MixUp-augmented images)
Optimization	Optimizer	AdamW [42]
	Learning rate	$1\times 10^{-4}$ for Stage 1 supervised contrastive training; $5\times 10^{-5}$ for Stage 2 joint fine-tuning
	Weight decay	$1\times 10^{-4}$
	Scheduler	Linear warm-up followed by cosine annealing warm restarts [43]; 5 warm-up epochs in Stage 1 and 3 warm-up epochs in Stage 2
	Early stopping	Patience = 15 epochs during Stage 2
	Gradient clipping	Maximum gradient norm = 2.0
Inference	Test-time augmentation	7 variants per test image

**Table 4:** Cumulative ablation study design used to evaluate the additional training and feature-refinement components in the proposed framework. Multi-scale feature extraction and class-weighted focal loss were retained in all configurations.

Experiment	CBS	TTA7	CBAM	MixUp	LS	SupCon
Exp1 (Baseline)	–	–	–	–	–	–
Exp2	Yes	–	–	–	–	–
Exp3	Yes	Yes	–	–	–	–
Exp4	Yes	Yes	Yes	–	–	–
Exp5	Yes	Yes	Yes	Yes	–	–
Exp6	Yes	Yes	Yes	Yes	Yes	–
Exp7	Yes	Yes	Yes	Yes	Yes	Yes

**Table 5:** SMIDS ablation results under ImageNet initialization and Hi-LabSpermMorpho domain-specific pretraining. Results are reported as average accuracy (%).

Exp.	1× ImageNet	1× Hi-Lab	5× ImageNet	5× Hi-Lab
Exp1	91.03	91.63	90.53	91.33
Exp2	91.23	91.97	90.33	90.93
Exp3	91.00	91.23	91.37	91.60
Exp4	91.30	91.57	90.53	91.47
Exp5	91.10	91.50	91.37	91.53
Exp6	90.77	91.93	91.57	91.73
Exp7	91.77	92.20	92.13	92.57

**Table 6:** Paired comparison between Exp7 and Exp1 using fold-level SMIDS accuracies. Δ: Exp7 − Exp1 (percentage points). CI and *p*-values were obtained with the corrected resampled *t*-test; Holm-adjusted *p*-values account for the four exploratory comparisons.

Setting	Fold-Level Δ (F1–F5)	Mean Δ [95% CI]	*p* (Corrected)	*p* (Holm)
1× ImageNet	+0.33, +0.67, +0.50, +0.34, +1.83	+0.73 [−0.44, +1.90]	0.157	0.313
1× Hi-Lab	+0.67, +2.00, +0.34, −0.33, +0.17	+0.57 [−1.06, +2.20]	0.388	0.388
5× ImageNet	+1.84, +1.50, +1.67, +1.50, +1.50	+1.60 [+1.32, +1.89]	<0.001	<0.001
5× Hi-Lab	+1.34, +0.67, +1.50, +1.17, +1.50	+1.24 [+0.59, +1.88]	0.006	0.018

**Table 7:** Design alternatives and component-removal experiments using Hi-LabSpermMorpho pretraining, 5× augmentation, and an identical training budget. Min denotes the lowest fold accuracy. Δ and *p* values (corrected resampled *t*-test) are reported relative to the full framework. These comparisons are exploratory; because none of the differences was statistically significant even without adjustment, multiple-comparison correction would not alter the conclusions. The final-stage-only variant was additionally evaluated with ImageNet initialization, achieving 91.93 ± 0.77% accuracy (Min: 90.67%), compared with 92.13 ± 0.62% (Min: 91.00%) for the full framework; this difference was not statistically significant (p=0.62).

Configuration	Mean ± SD (%)	Min (%)	Δ	*p*
Full framework	92.57 ± 0.35	92.00	–	–
Final-stage representation only	92.63 ± 0.65	91.67	+0.07	0.81
Middle + late stages	92.40 ± 0.50	91.67	−0.17	0.35
No early-stage freezing	92.57 ± 0.59	91.67	0.00	1.00
Without CBAM	92.53 ± 0.54	91.50	−0.03	0.92
Without class-balanced sampling	92.60 ± 0.87	91.17	+0.03	0.94
Without MixUp	92.50 ± 0.85	90.83	−0.07	0.88
Without label smoothing	92.50 ± 0.51	91.67	−0.06	0.86
Cross-entropy instead of focal loss	92.67 ± 0.28	92.33	+0.10	0.67

**Table 8:** Five-fold SMIDS classification results obtained using the complete Exp7 framework under different augmentation and initialization settings. Results are reported as accuracy (%).

Initialization	Augmentation	Fold 1	Fold 2	Fold 3	Fold 4	Fold 5	Average	Std.
Hi-LabSpermMorpho	1×	92.32	93.18	92.67	90.67	92.17	92.20	0.84
Hi-LabSpermMorpho	5×	92.32	92.85	92.83	92.00	92.83	92.57	0.35
Hi-LabSpermMorpho	10×	92.82	93.34	92.33	92.17	93.00	92.73	0.43
ImageNet	1×	91.65	91.35	92.67	90.67	92.50	91.77	0.74
ImageNet	5×	92.82	92.18	92.50	91.00	92.17	92.13	0.62
ImageNet	10×	92.49	92.68	92.83	90.67	91.83	92.10	0.79

**Table 9:** Confusion-matrix-based performance metrics for the complete Exp7 framework.

Initialization	Augmentation	Accuracy (%)	Macro Precision (%)	Macro Recall (%)	Macro F1-Score (%)
Hi-LabSpermMorpho	1×	92.20	92.39	92.23	92.29
Hi-LabSpermMorpho	5×	92.57	92.72	92.59	92.63
Hi-LabSpermMorpho	10×	92.73	92.84	92.77	92.79
ImageNet	1×	91.77	91.98	91.79	91.86
ImageNet	5×	92.13	92.29	92.16	92.20
ImageNet	10×	92.10	92.23	92.13	92.17

**Table 13:** Class-specific performance of the best configuration (ConvNeXt-Tiny with Hi-LabSpermMorpho pretraining and 10× augmentation), computed from the out-of-fold predictions of all 3000 test images. 95% confidence intervals: Wilson intervals for recall and precision; bootstrap (5000 resamples) for F1-score, balanced accuracy, and macro F1-score.

Class	Recall (%)	Precision (%)	F1-Score (%)	*n*
Abnormal sperm	89.45 [87.40, 91.20]	90.17 [88.17, 91.87]	89.81 [88.36, 91.14]	1005
Non-sperm	96.00 [94.57, 97.06]	97.80 [96.67, 98.56]	96.89 [96.14, 97.68]	974
Normal sperm	92.85 [91.10, 94.28]	90.54 [88.62, 92.17]	91.68 [90.39, 92.84]	1021
Balanced accuracy: 92.77 [91.83, 93.67]; macro F1-score: 92.79 [91.85, 93.68]				

## Data Availability

The SMIDS dataset used as the target benchmark dataset in this study is publicly available from the source reported by Ilhan et al. [26]. The Hi-LabSpermMorpho dataset used for domain-specific pretraining is publicly available in an online repository: https://github.com/Yildiz-Hi-Lab/Hi-LabSpermMorpho (accessed on 11 October 2026).

## Abbreviations

The following abbreviations are used in this manuscript:

AdamW	Adam optimizer with decoupled weight decay
BEiT	BERT Pre-Training of Image Transformers
CBAM	Convolutional Block Attention Module
CBS	Class-Balanced Sampling
CNN	Convolutional Neural Network
DeiT	Data-Efficient Image Transformer
GAP	Global Average Pooling
GELU	Gaussian Error Linear Unit
lr	Learning Rate
LS	Label Smoothing
MLP	Multi-Layer Perceptron
RGB	Red–Green–Blue
SMIDS	Sperm Morphology Image Dataset for Segmentation
SupCon	Supervised Contrastive Learning
TTA	Test-Time Augmentation
